# Crosstalk of hepatocyte nuclear factor 4a and glucocorticoid receptor in the regulation of lipid metabolism in mice fed a high-fat-high-sugar diet

**DOI:** 10.1186/s12944-022-01654-6

**Published:** 2022-05-25

**Authors:** Hong Lu, Xiaohong Lei, Rebecca Winkler, Savio John, Devendra Kumar, Wenkuan Li, Yazen Alnouti

**Affiliations:** 1 Department of Pharmacology, SUNY Upstate Medical University, Syracuse, NY 13210 USA; 2 Department of Medicine, SUNY Upstate Medical University, Syracuse, NY 13210 USA; 3 Department of Pharmaceutical Sciences, University of Nebraska Medical Center, Omaha, NE 68198 USA

**Keywords:** HNF4α, heterozygote, knockout, hyperlipidemia, fatty liver, GR, PPARα, LXR, SREBP-1C, high-fat diet

## Abstract

**Background:**

Hepatocyte nuclear factor 4α (HNF4α) and glucocorticoid receptor (GR), master regulators of liver metabolism, are down-regulated in fatty liver diseases. The present study aimed to elucidate the role of down-regulation of HNF4α and GR in fatty liver and hyperlipidemia.

**Methods:**

Adult mice with liver-specific heterozygote (HET) and knockout (KO) of HNF4α or GR were fed a high-fat-high-sugar diet (HFHS) for 15 days. Alterations in hepatic and circulating lipids were determined with analytical kits, and changes in hepatic mRNA and protein expression in these mice were quantified by real-time PCR and Western blotting. Serum and hepatic levels of bile acids were quantified by LC-MS/MS. The roles of HNF4α and GR in regulating hepatic gene expression were determined using luciferase reporter assays.

**Results:**

Compared to HFHS-fed wildtype mice, HNF4α HET mice had down-regulation of lipid catabolic genes, induction of lipogenic genes, and increased hepatic and blood levels of lipids, whereas HNF4α KO mice had fatty liver but mild hypolipidemia, down-regulation of lipid-efflux genes, and induction of genes for uptake, synthesis, and storage of lipids. Serum levels of chenodeoxycholic acid and deoxycholic acid tended to be decreased in the HNF4α HET mice but dramatically increased in the HNF4α KO mice, which was associated with marked down-regulation of cytochrome P450 7a1, the rate-limiting enzyme for bile acid synthesis. Hepatic mRNA and protein expression of sterol-regulatory-element-binding protein-1 (SREBP-1), a master lipogenic regulator, was induced in HFHS-fed HNF4α HET mice. In reporter assays, HNF4α cooperated with the corepressor small heterodimer partner to potently inhibit the transactivation of mouse and human SREBP-1C promoter by liver X receptor. Hepatic nuclear GR proteins tended to be decreased in the HNF4α KO mice. HFHS-fed mice with liver-specific KO of GR had increased hepatic lipids and induction of SREBP-1C and PPARγ, which was associated with a marked decrease in hepatic levels of HNF4α proteins in these mice. In reporter assays, GR and HNF4α synergistically/additively induced lipid catabolic genes.

**Conclusions:**

induction of lipid catabolic genes and suppression of lipogenic genes by HNF4α and GR may mediate the early resistance to HFHS-induced fatty liver and hyperlipidemia.

**Graphical abstract:**

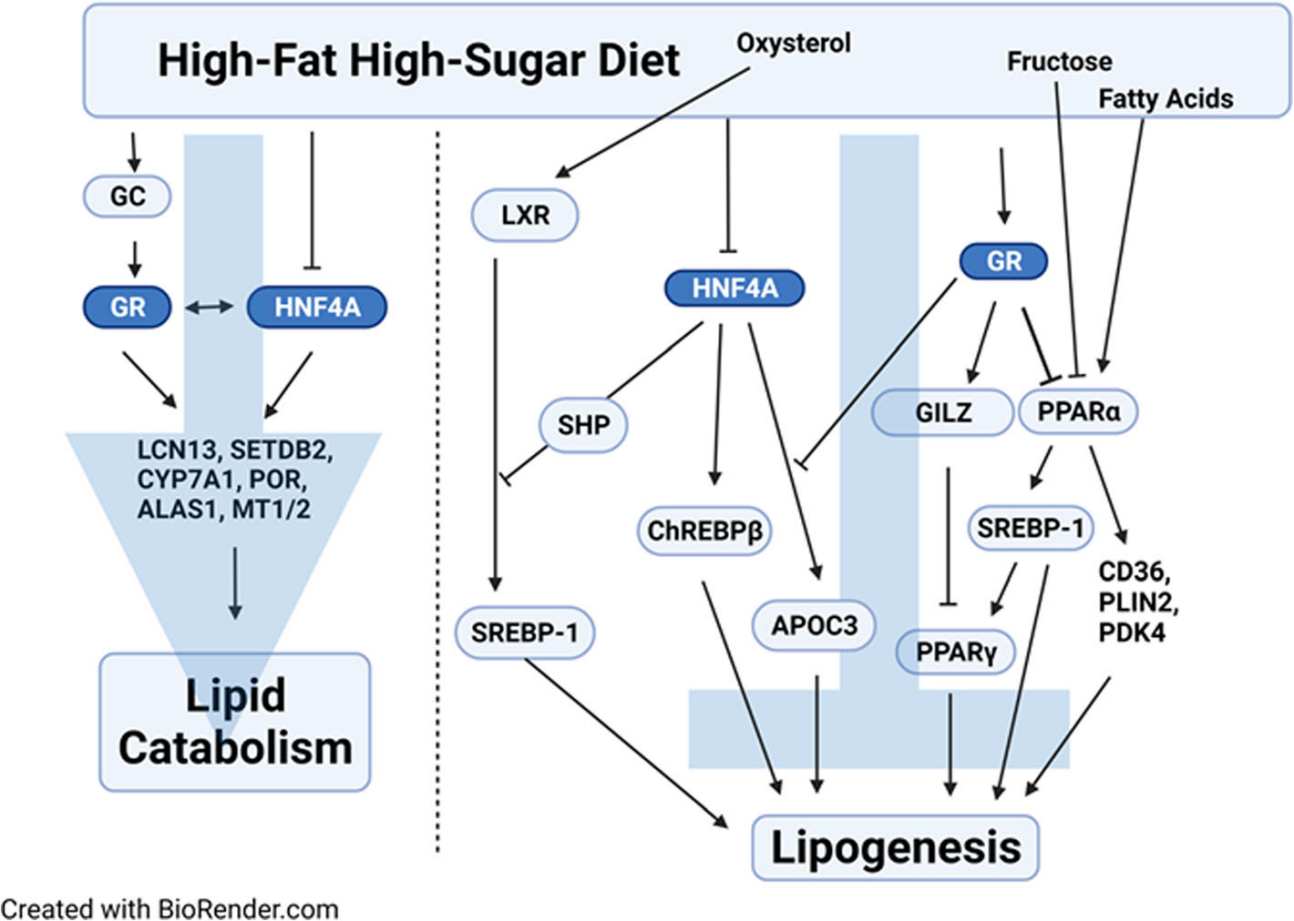

**Supplementary Information:**

The online version contains supplementary material available at 10.1186/s12944-022-01654-6.

## Background

In modern society, the synergy between excessive psychological stress and overeating high-fat and high-sugar diets (HFHS) propels a pandemic of non-alcoholic fatty liver disease (NAFLD) which has now replaced viral hepatitis as the most common chronic liver disease [1]. It is well known that high-fat diet (HFD) does not cause fatty liver until after long-term feeding [2]. A poorly understood knowledge gap is the molecular mechanism of early resistance of the liver to HFD/HFHS-induced steatosis and how the resistance is compromised over time; bridging this key knowledge gap will help discover novel preventive and therapeutic strategies for NAFLD.

Hepatocyte nuclear factor 4α (HNF4α) is a master regulator of liver metabolism and lipid homeostasis via crosstalk with diverse extracellular and intracellular signaling pathways to regulate hepatic nutrient metabolism [3]. Hepatic HNF4α expression is markedly decreased in diabetes and NAFLD which are commonly associated with hyperlipidemia [4–6]. Paradoxically, adult chow-fed mice with liver-specific knockout (KO) of HNF4α have fatty liver but striking hypolipidemia, and these HNF4α KO mice are protected from atherosclerosis [4]. Thus, suppression of hepatic HNF4α has been proposed to prevent atherosclerosis [4]. However, the marked hypolipidemia in chow-fed adult HNF4α KO mice sharply contrasts with the hyperlipidemia in patients and animal models with NAFLD/non-alcoholic steatohepatitis (NASH) in which HNF4α is partially lost [7, 8], a condition that may be better mimicked in mice with hetero-deficiency of HNF4α. The most common cause of death among patients with NAFLD is coronary artery disease (CAD) [9]. In contrast to hyperlipidemia in NAFLD, hypolipidemia often occurs in patients with end-stage liver diseases, such as cirrhosis and liver cancer [10], in which the loss of functional HNF4α is more marked [11] and may be better mimicked by the use of HNF4α KO mice. Thus, there is a key knowledge gap regarding the gene-dosage-dependent roles of the partial and total loss of HNF4α in regulating hepatic lipid metabolism, circulating lipids, and CAD.

Because diet intake has a critical role in modulating hepatic lipid metabolism and circulating lipid profiles, the purpose of this study was to uncover how interactions between gene-dosage-dependent HNF4α deficiency and HFD may differentially alter hepatic gene expression and lipid metabolism. In previous studies [12, 13], the adult HNF4a KO mice were fed a low-fat chow. Interestingly, the present study found that HFHS-fed HNF4α heterozygote (HET) mice had elevated hepatic and blood levels of cholesterol and triglycerides. Results from further mechanistic studies suggest that HNF4α has a key role in regulating hepatic lipid metabolism and circulating lipids by inducing lipid catabolic genes and suppressing lipogenic genes.

Biosynthesis of BAs from cholesterol is a major pathway for the catabolism of cholesterol, whereas biliary excretion of BAs is essential for BA and cholesterol elimination and intestinal absorption of lipids (Supplemental Figure 1https://figshare.com/s/008a9f1d4b7834a8f6c4). In the liver, the classical pathway of CYP7A1-CYP8B1 prefers the biosynthesis of hydrophilic cholic acid (CA), and the alternative pathway of CYP27A1-CYP7B1 prefers the hydrophobic chenodeoxycholic acid (CDCA). Both CYP27A1 and CYP7B1 are expressed in various tissues, whereas CYP7A1 and CYP8B1 are liver-specific [14]. In rodents, the hydrophobic CDCA is largely converted to the highly hydrophilic muricholic acid (MCA) by CYP2C70, resulting in a much more hydrophilic BA pool than in humans [15]. Additionally, CDCA can be detoxified by CYP3A to more hydrophilic hyocholic acid (HCA) [16]. The hydrophilic ursodeoxycholic acid (UDCA), a primary BA in the bear, is a minor form in mice. In the intestine, CA and CDCA are metabolized by bacteria to cytotoxic deoxycholic acid (DCA) and lithocholic acid (LCA), respectively. Most LCA can be efficiently sulfated and excreted from the intestine, whereas unconjugated DCA is reabsorbed in the large intestine and transported back to the liver, where it can be conjugated [17]. In rodents, the taurine (T) conjugated DCA (T-DCA) can be converted back to T-CA via 7-hydroxylation by Cyp2a12 [18]. Hyodeoxycholic acid (HDCA), generated by bacterial C-6 hydroxylation of LCA or dehydroxylation of HCA in the small intestine, is an LXRα agonist [19] (Supplemental Figure 1https://figshare.com/s/008a9f1d4b7834a8f6c4). Approximately 95% of BAs, consisting of mainly CA, DCA, and CDCA in humans and CA and α/β-MCA in mice, are reabsorbed via enterohepatic circulation. Only ~5% of BAs are synthesized *de novo* from cholesterol daily [20]. In this study, hepatic partial and complete deficiency of HNF4α in HFHS-fed mice distinctly affect the hepatic expression of BA synthetic and metabolic genes, resulting in distinct blood and hepatic BA profiles.

Consumption of HFHS “comfort foods” is associated with elevated circulating glucocorticoids (GCs) [21]. Literature suggests a hepatic deficiency of glucocorticoid receptor (GR) in human NAFLD [22, 23]. Currently, the role of hepatic GR in fatty liver is still controversial [24–26]. This study found that hepatic nuclear GR proteins tended to be decreased by HNF4α deficiency in HFHS-fed mice. In parallel, HFHS-fed GR KO mice had marked down-regulation of HNF4α, elevated hepatic levels of cholesterol and triglycerides, and induction of key lipogenic genes. GR and HNF4α cooperatively activated promoters of key lipid catabolic genes, and HNF4α and GR antagonized the induction of lipogenic genes by liver X receptor (LXR) and peroxisome proliferator-activated receptor alpha (PPARα), two master regulators of lipid metabolism [27, 28]. Thus, GR and HNF4α cooperatively protect against HFHS-induced fatty liver and hyperlipidemia.

## Methods

### Generation and treatment of mice

Mice with tamoxifen-inducible liver-specific KO and HET of HNF4α were generated by crossing HNF4α floxed mice [12] with mice carrying tamoxifen-inducible estrogen-receptor-fused Cre under the control of an albumin promoter (SA^+/CreERT2^) [29]. Adult (8-week-old) male WT (Cre/-), HET (HNF4α fl/+, Cre/+), and KO (HNF4α fl/fl, Cre/+) mice were administered tamoxifen (T5648, Sigma, 5 mg/kg IP in corn oil) once daily for 2 d. The day after the 2nd tamoxifen treatment, mice were fed a HFHS that contained a high-fat diet (HFD, 60% fat kcal, #D12492, Research Diets) and high-sugar drinking water with 42 g/L of sugar (55% fructose/45% sucrose) [30]. All mice were allowed water and feed *ad libitum* and sacrificed 15 d or 6 weeks (*N*=6 per group) after diet treatments without pre-fasting to collect liver and blood samples.

Mice with tamoxifen-inducible liver-specific KO of GR were generated by crossing GR floxed mice (Stock# 021021, Jackson Laboratory) [31] with the SA^+/CreERT2^ mice [29]. Adult (8-10-week-old) male WT (GR fl/fl, Cre/-) and GR KO (GR fl/fl, Cre/+) mice (*N*=6 per group) were administered tamoxifen (T5648, Sigma, 5 mg/kg IP in corn oil) once daily for 2 d. The day after the 2nd tamoxifen treatment, mice were fed HFHS [30]. All mice were allowed water and feed *ad libitum* and sacrificed 15 d after HFHS feeding, without pre-fasting to collect liver and blood samples.

Blood glucose was determined by Care Touch Blood Glucose Meter using tail-tip blood. After anesthetization by IP injection of Ketamine (100 mg/kg) + Xylazine (10 mg/kg), blood samples were collected by orbital bleeding, followed by harvest of livers. Regarding euthanasia, mice were deceased quickly after liver harvest. Liver tissues were snap-frozen in liquid nitrogen upon collection and stored at -80 °C until use. To prepare serum samples, the clotted blood samples were centrifuged at 8000 rpm for 10 min. All animals received humane care and all animal procedures in this study were approved by the *Institutional Animal Care and Use Committee* (IACUC) of the SUNY Upstate Medical University.

### Construction of reporter and expression vectors

The PCR primers were synthesized by Integrated DNA Technologies (IDT). Total genomic DNA extracted from liver mouse and HEK293 cells (ATCC) were used as templates for PCR-cloning of the mouse/human promoter/intron fragments into the KpnI/MluI sites of the pGL3 basic luciferase reporter vector (Promega). The glutamic acid (E) to lysine (K) at 363 (E363K) mutant of pCDNA3-HNF4A2 was generated using Q5® Site-Directed Mutagenesis Kit (New England Biolabs). The pcDNA3- small heterodimer partner (SHP)-Flag vector for SHP was synthesized by GenScript. The primers and sequence information of all reporter constructs is provided in Supplemental Table S1 (https://figshare.com/s/650749d0f09c1c5d4b2e). All the constructed vectors were verified by sequencing.

### Transient transfection and dual-luciferase assay

Human embryonic kidney 293 (HEK293) cells and the HepG2/C3A human hepatocellular carcinoma cells (ATCC) were cultured with MEM and EMEM medium (Corning), respectively supplemented with 10% fetal calf serum. Twenty-four hours after seeding, transfection was conducted using Lipofectamine 2000 (Invitrogen), following the manufacturer's protocol. In the 96-well-plate, each well *was* transfected with firefly luciferase vectors, the control renilla luciferase vector pRL-CMV, and/or the mammalian expression vectors for human HNF4A2 (#31100, Addgene), LXRα [28], GR (#15534, Addgene), SHP, and/or the pCMX backbone vector to add up the total DNA vectors to 100 ng. Dexamethasone (#11015, Cayman Chemical) (10 nM) was added 1 h after cells were transfected with the GR expression vector. Twenty-four hours after transfection, cells were harvested for dual-luciferase assay using Dual-Glo™ luciferase assay system (Promega) and GloMax Luminometer (Promega), following the manufacturer’s protocol. The ratios of firefly/renilla luciferase activities were calculated as the normalized reporter activity, with the control values set at 1.0.

### Western blotting

A small piece of frozen liver was homogenized in hypotonic buffer (20 mM HEPES, 5 mM potassium acetate, 0.5 mM MgCl_2_, pH 7.8 with KOH) with protease and phosphatase inhibitors (Pierce Protease and Phosphatase Inhibitor Mini Tablets, Product # A32959, Thermo Scientific, and/or Halt Protease & Phosphatase Inhibitor Single-Use Cocktail, Product # 1861280, Thermo Scientific). Nuclei were pelleted with slow (1500 g for 5 minutes) centrifugation(s). Supernatants were used for cytoplasmic fractions, and pellets were digested in RIPA buffer (10 mM Tris-HCl, 1 mM EDTA, 0.5 mM EGTA, 1% Triton X-100, 0.1% sodium deoxycholate, 0.1% SDS, 140 mM NaCl, and water to 100 mL (http://cshprotocols.cshlp.org/content/2006/4/pdb.rec10617.short), final pH 7.5-8.0) with protease and phosphatase inhibitors for nuclear fractions. Proteins in cytosolic or nuclear extracts from mouse livers were resolved in sodium dodecyl sulphate-polyacrylamide gel electrophoresis. Western blotting quantification of proteins was conducted with primary antibodies as follows: HNF4α (#3113, Cell Signaling), GR (D6H2L XP, # 12041, Cell Signaling), acetyl-CoA carboxylase (ACC, #3676, Cell Signaling), fatty acid synthase (FASN, # 3180, Cell Signaling), SREBP-1 (MABS1987, Sigma), PPARα (PA1412, Boster Bio), ATP Citrate Lyase (ACLY, #13390, Cell Signaling), phosphorylated epidermal growth factor receptor (p-EGFR, sc-12351-R, Santa Cruz Biotechnology), phosphorylated (Tyr705) signal transducer and activator of transcription 3 (pSTAT3, #9145T, Cell Signaling). An aliquot of total cytosolic or nuclear proteins was mixed with loading buffer (0.2 M Tris-HCl, 0.4 M DTT, 8% SDS, 6 mM bromophenol blue, 4.3 M glycerol), was denatured, and 30 ug protein was loaded into each lane of Mini-PROTEAN TGX Stain-Free Precast Gels (Bio-Rad) for sodium dodecyl sulphate-polyacrylamide gel electrophoresis (SDS-PAGE). Prior to blocking in 3% milk or BSA, blots were soaked in hydrogen peroxide to inactivate endogenous peroxidases [32]. Primary antibodies were revealed with HRP-conjugated secondary antibodies (Anti-rabbit IgG, #7074, Cell Signaling and/or HRP Conjugated AffiniPure Goat Anti-Rabbit IgG (H+L), BA1054-0.5, Boster) and ECL Western Blotting Substrate (W1015, Promega). Target proteins were normalized to the loaded total proteins in each lane. ChemiDoc^TM^ XRS+System and Image Lab version 6.1.0 build 7 Standard Edition from Bio-Rad Laboratories, Inc. was used for quantification. A multichannel image was set up with the image of the total protein blot and the image of the bands to be analyzed. "Normalization Channel" was set to "Stain Free Blot". Lanes were delineated, and bands of interest were marked. Background subtraction was done and visualized for each image separately utilizing the "Lane Profile" icon. A report was generated, and data were collected. For Western blotting with too many samples to fit on a single blot, fold changes of bands in the HNF4α HET and KO groups relative to the WT group were determined for each blot separately. The resulting data were then combined from both blots for graphing and statistics.

### Real-time PCR

Total RNAs from mouse livers were isolated by RNA-STAT60 (Tel-Test) and quantified by NanoDrop^TM^ spectrophotometer (Thermo Scientific). For real-time PCR, 1 μg of RNA was reverse transcribed using the High-Capacity RNA-to-cDNA^TM^ Kit (Applied Biosystems®, life technologies) for cDNA synthesis, following the manufacturer's instructions. iQ™ SYBR® Green Supermix (Bio-Rad) was applied to quantify mRNAs using MyiQ2™ Two-Color Real-Time PCR Detection System (Bio-Rad). Alternatively, total RNAs from mouse livers were isolated by RNA-STAT60 (Tel-Test) and quantified by NanoDrop^TM^. RNA was reverse transcribed using iScript^TM^ cDNA Synthesis Kit (Bio-Rad), following which qPCR was performed using iTaq™ Universal SYBR® Green Supermix (Bio-Rad) using Bio-Rad’s CFX Maestro (Version: 4.1.2433.1219.). The amounts of mRNA were calculated using the comparative CT method, which determines the amount of target gene normalized to peroxiredoxin 1 (Prdx1) or phosphoglycerate kinase 1 (Pgk1), two of the most stable housekeeping genes [33, 34]. The specificity of the real-time PCR primers was verified using the no-reverse-transcriptase control. Sequences of real-time PCR primers (synthesized by IDT) were listed in Supplemental Table S2 (https://figshare.com/s/52b2fd910184cc82d322).

### Determination of lipids in mouse liver

Frozen liver tissues were homogenized in buffer containing 18 mM Tris, pH 7.5, 300 mM mannitol, 10 mM EGTA, and 0.1 mM phenylmethylsulfonyl fluoride [35]. Liver homogenates were mixed with chloroform:methanol (2,1) and incubated overnight at room temperature with occasional shaking. The next day, H_2_O was added, vortexed, and centrifuged at 3000 *g* for 5 min. The lower lipid phase was collected and concentrated by a speed vacuum concentrator. The lipid pellets were dissolved in a mixture of 270 μl of isopropanol and 30 μl of Triton X-100 to determine triglycerides and total cholesterol using commercial analytical kits (Pointe Scientific, Inc, Canton MI).

### Determination of blood levels of glucose, insulin, cholesterol, free fatty acids, and ketone bodies

Blood levels of glucose were quantified with Precision Xtra Glucose Monitor (#179837, Bound Tree Medical, LLC, Chicago, IL). Serum levels of insulin were determined with an ELISA kit (#90080, Crystal Chem USA, Downers Grove, IL). Serum levels of total cholesterol, high-density lipoprotein (HDL) cholesterol, and low-density lipoprotein (LDL)/very-low-density lipoprotein (VLDL) cholesterol were quantified with HDL and LDL/VLDL assay kit (EHDL-100, Bioassay Systems). Serum levels of free fatty acids were analyzed with EnzyChrom™ Free Fatty Acid Assay Kit (EFFA-100, Bioassay Systems). Serum levels of β-hydroxybutyrate were quantified with a colorimetric assay kit (#700190, Cayman Chemical, Ann Arbor, MI).

### Bile acid quantification

Bile acids in liver and serum in HFHS-fed wild-type, HNF4α HET, and HNF4α KO mice were quantified by liquid chromatography-tandem mass spectrometry (LC-MS/MS), as described previously with some modifications [36, 37]. Briefly, a Waters ACQUITY ultra-performance LC system (Waters, Milford, MA, USA) coupled to a 4000 Q TRAP® quadrupole linear ion trap hybrid MS with an electrospray ionization source (Applied Biosystems, MDS Sciex, Foster City, CA, USA) was used. The following MS source settings were used: ion spray voltage, −4500 V; temperature, 550°C; curtain gas, 10; gas-1, 40; gas-2 40 (arbitrary units); collision gas pressure, high; Q1/Q3 resolution, unit; and interface heater, on. The mobile phase consisted of 7.5 mm ammonium bicarbonate, adjusted to pH 9.0 using ammonium hydroxide (mobile phase A) and 30% acetonitrile in methanol (mobile phase B) at a total flow rate of 0.2 mL min^−1^. The gradient profile was held at 52.5% mobile phase B for 12.75 minutes, increased linearly to 68% in 0.25 minutes, held at 68% for 8.75 minutes, increased linearly to 90% in 0.25 minutes, held at 90% for 1 minute, and finally brought back to 52.5% in 0.25 minutes followed by 4.75 minutes re-equilibration (total run time of 28 minutes per sample). For the preparation of calibration curves, blank matrices were obtained by charcoal stripping as described previously [36, 37]. Eleven-point calibration curves were prepared by spiking 10 uL of appropriate standard solution into 50 uL stripped serum and 100 uL stripped liver homogenate at final concentrations ranging from 1 to 1000 ng mL^−1^.

For the preparation of serum samples, 50 uL of serum samples were spiked with 10 uL of internal standard, 1 mL of ice-cold alkaline acetonitrile (5% NH_4_OH) was added, and samples were vortexed. Samples were then centrifuged at 16 000 *g* for 10 minutes and the supernatants were aspirated, evaporated under vacuum, and reconstituted in 100 uL of 50% MeOH solution. For liver samples, approximately 100 mg of the liver was homogenized in 4 volumes of water. A 100 uL of liver homogenate was spiked with 10 uL IS, and 2 mL of ice-cold alkaline ACN was added. Samples were vortexed, shaken continuously for 15 min, and then centrifuged at 16000×g for 10 min. The supernatant was aspirated and the pellet was extracted with another 1 mL of ice-cold alkaline ACN. Supernatants from the 2 extraction steps were pooled, evaporated, and reconstituted in 100 uL of 50% MeOH.

The hydrophobicity index (HI) of bile acids was calculated by the method of Heuman [38].

### Statistical analysis

An N = 6-7 mice per group was used in this study based on power analysis and the past studies of HNF4α KO mice [12]. Samples were randomly picked for analysis. Before statistical analysis, data were analyzed for normality using the “Identify Outliers” function in GraphPad Prism 9.1. One wildtype mouse had very high serum and hepatic levels of BAs and was determined as an outlier and removed from statistical analysis. Except for values in boxplots, all values were expressed as mean ± S.E. For comparison of the two groups, the two-tailed student’s t-test was used to determine the statistical difference, which was set at *P <* 0.05. For multiple comparisons with the wildtype mice in animal studies, analysis of variance (ANOVA) was performed, followed by the Dunnett T3 test to correct for multiple comparisons using statistical hypothesis testing, with significance set at *P <* 0.05. In the dual-luciferase reporter assays, the Tukey test was conducted to correct for multiple comparisons between every two groups, with significance set at *P <* 0.05. Magnitudes of change were calculated by dividing the control group average by the experimental group average.

For Western blotting, after quantifying band densities each value was divided by the average of the WT values for that particular antibody and blot as a way to graph values relative to WT and as a way to combine data from two different blots (see Fig. 6). Graphing and statistics were then performed in GraphPad Prism 9.3.1 (471) for Windows, GraphPad Software, San Diego, California USA, www.graphpad.com. For HNF4α Western blotting with 3 groups (WT, HET, and KO), normality was checked using Shapiro-Wilk test. Data passing normality testing were analyzed via Brown-Forsythe and Welch ANOVA tests followed by Dunnett's T3 multiple comparisons. Data not passing normality testing were analyzed with nonparametric Kruskal-Wallis ANOVA and multiple comparisons. For GR KO Western blotting with 2 groups (WT vs KO), normality was checked using the Shapiro-Wilk test. Data were then analyzed with Welch's t-test when normality was not significant, and Kolmogorov-Smirnov when normality was deemed significant. Comparisons were made between WT vs. HET and WT vs. KO, and *p* values less than 0.05 were marked with a single asterisk.

## Results

### Effects of HFHS on blood lipid profiles in HNF4α HET and KO mice

**Fig. 1:**
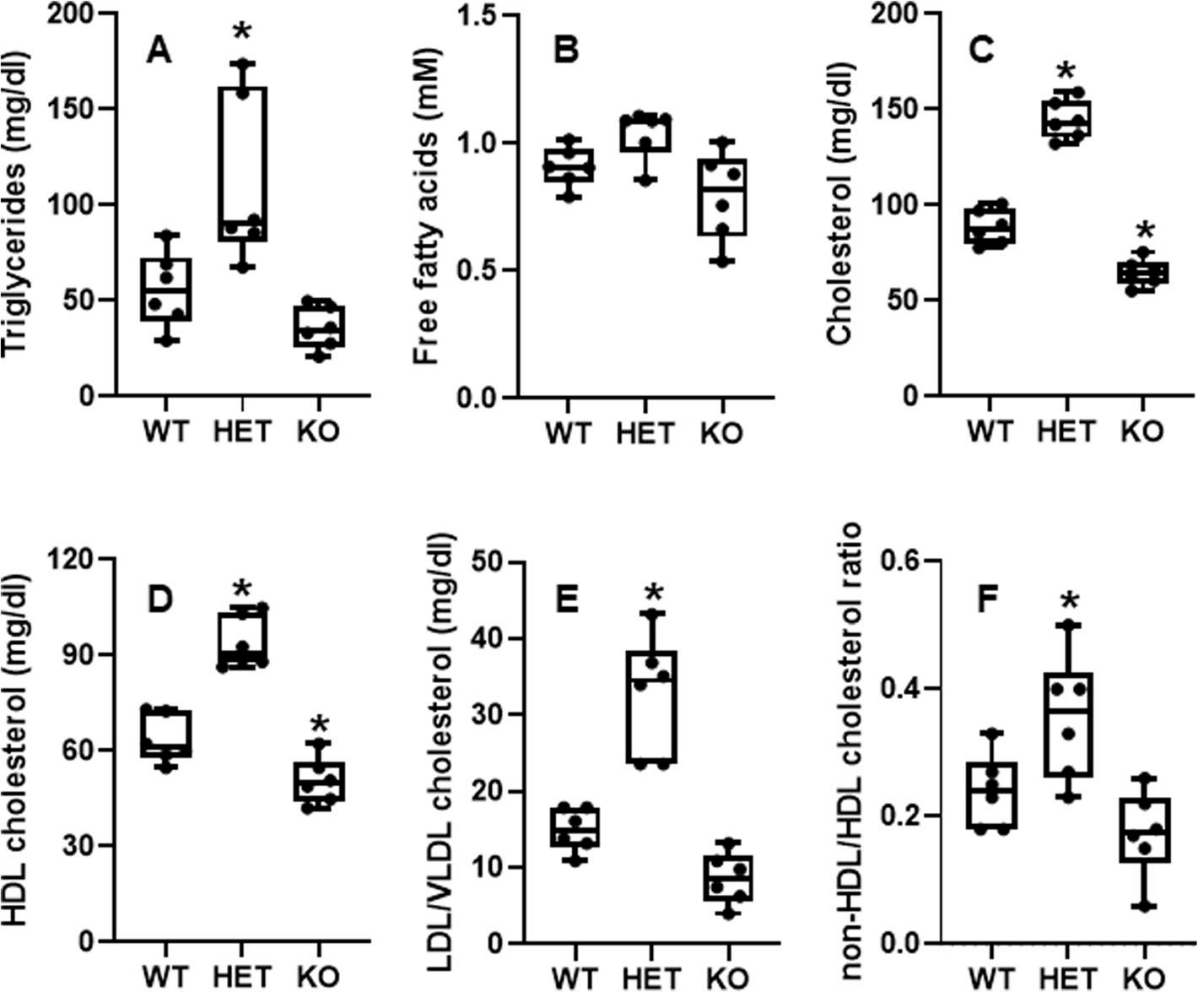
Blood levels of (**A**) triglycerides, (**B**) free fatty acids, (**C**) total cholesterol, (**D**) HDL cholesterol, (**E**) LDL/VLDL cholesterol, and (**F**) ratio of non-HDL/HDL cholesterol in adult male wildtype (WT), HNF4α heterozygote (HET), and HNF4α knockout (KO) mice. Mice were fed 15 d with high-fat-high-sugar diet (HFHS) (*N*=6 per group). In the box plots, the end of the lower whisker is the minimum value, the boundary of the box closest to zero indicates the 25th percentile, a black line within the box marks the median, the boundary of the box farthest from zero indicates the 75th percentile, and the end of the upper whisker is the maximum value. * *P <* 0.05 versus WT mice

After being fed HFHS for 15 d, HNF4α HET mice had 93% higher triglycerides (TG) (Fig. 1A), 14% higher free fatty acids (Fig. 1B), 63% higher total cholesterol (Fig. 1C), 48% higher HDL cholesterol (Fig. 1D), and 120% higher LDL/VLDL cholesterol (Fig. 1E) than WT mice, whereas HNF4α KO mice had mild hypolipidemia, namely 34% lower TG (Fig. 1A), 13% lower free fatty acids (Fig. 1B), 27% lower total cholesterol (Fig. 1C), 20% lower HDL cholesterol (Fig. 1D), and 42% lower LDL/VLDL cholesterol (Fig. 1E) than WT mice. Interestingly, the ratio of non-HDL/HDL cholesterol increased in the HET but tended to decrease in the KO mice (Fig. 1F). Further studies were focused on understanding the distinct gene-dosage-dependent changes in lipid metabolism in the HFHS-fed HNF4α HET and KO mice.

### Changes in liver/body weight and blood levels of glucose and insulin in HFHS-fed mice

Fifteen days after HFHS feeding, WT and HNF4α HET mice tended to gain weight, whereas the KO mice tended to lose weight (Table 1). The liver/body weight ratio was 18% and 47% higher in the HET and KO mice, respectively, compared to WT mice. Blood levels of glucose remained unchanged in HET mice but decreased by 35% in KO mice. Consistent with changes in blood glucose, serum levels of insulin remained unchanged in HET but decreased by 36% in KO mice. These data suggest enhanced hepatic insulin signaling in HFHS-fed HNF4α KO mice. Interestingly, blood levels of ketone bodies, determined by β-hydroxybutyrate, were 63% and 29% higher in the HET and KO mice (Table 1).

**Table 1:** Changes of body weight, liver/body weight as well as blood levels of glucose, insulin, and ketone bodies in adult male wildtype, HNF4α heterozygote, and HNF4α knockout mice fed the high-fat-high-sugar diet for 15 d

	Wildtype	Heterozygote	Knockout
Body weight (Day0) (g)	23.6 ± 0.6	25.0 ± 0.5	23.7 ± 0.5
Body weight (Day15) (g)	24.9 ± 0.9	25.7 ± 0.5	21.7 ± 1.0
Liver/body weight (g/100 g)	4.25 ± 0.11	5.00 ± 0.19 *	6.25 ± 0.21 *
Glucose (mg/dl)	199 ± 5	180 ± 7	130 ± 9 *
Insulin (nM)	0.21 ± 0.02	0.21 ± 0.06	0.13 ± 0.02 *
Ketone body (μM)	205 ± 8	333 ± 46 *	264 ± 11 *

*N*=6-7 per group, mean ± SE. * *P <* 0.05 versus wildtype mice

### Effects of HFHS on hepatic lipid profiles in HNF4α HET and KO mice

**Fig. 2:**
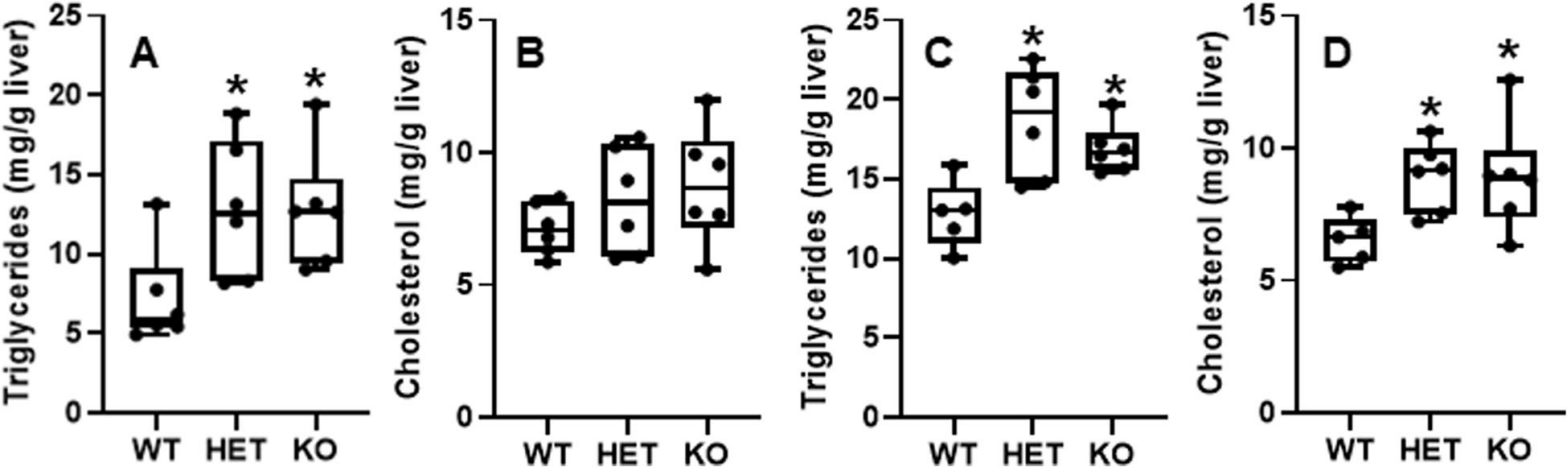
Hepatic lipids in adult male wildtype (WT), HNF4α heterozygote (HET), and/or HNF4α knockout (KO) mice. Mice were fed high-fat-high-sugar diet (HFHS) for 15 d (**A** & **B**) or 6 weeks (6 wk) (**C** & **D**). *N*=6 per group. In the box plots, the end of the lower whisker is the minimum value, the boundary of the box closest to zero indicates the 25th percentile, a black line within the box marks the median, the boundary of the box farthest from zero indicates the 75th percentile, and the end of the upper whisker is the maximum value. * *P <* 0.05 versus WT mice

After being fed the HFHS for 15 d, both the HET and KO mice had marked 1.4 and 1.2 fold increases in hepatic TG, respectively, compared to WT mice (Fig. 2A). Hepatic cholesterol tended to be higher in the HNF4α HET and KO mice (Fig. 2B). Compared to the 15-day HFHS diet, 6-week HFHS induced 1.4 fold greater increase in hepatic TG in the WT mice (Fig. 2C). The HNF4α HET mice had 46% higher TG (Fig. 2C) and 37% higher cholesterol (Fig. 2D) than WT mice 6 weeks after HFHS. The data demonstrate a dramatic increase in the fatty liver after partial loss of HNF4a, a condition that worsens over time. Thus, partial loss of HNF4a is likely a key mechanism of hepatic decompensation and gradual buildup of fatty liver with HFHS diet.

### Changes in hepatic histology

**Fig. 3:**
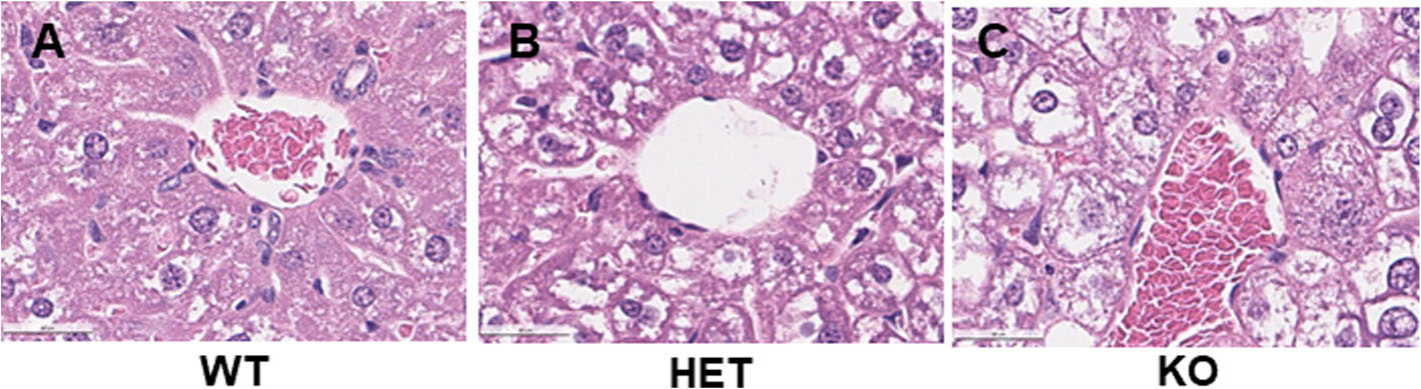
Liver histology in adult male wildtype (WT), HNF4α heterozygote (HET), and HNF4α knockout (KO) mice fed 15 d with high-fat-high-sugar diet (HFHS). H&E staining of paraffin embedded liver sections (5 μm and 400 × magnification). **A** WT; **B** HET; **C** KO

After being fed the HFHS for 15 d, compared to the WT mice (Fig. 3A), the HNF4α HET mice (Fig. 3B) had more vacuolization of hepatocytes. Moreover, similar to the chow-fed HNF4α KO mice [29], the HFHS-fed KO mice (Fig. 3C) had more marked enlargement and vacuolization of hepatocytes than the HET and KO mice. No obvious infiltration of inflammatory cells was observed in any of these livers.

### Changes in blood and hepatic levels of bile acids (BAs) in HNF4α HET and KO mice 15 d after HFHS feeding

#### Changes in blood levels of BAs

**Fig. 4:**
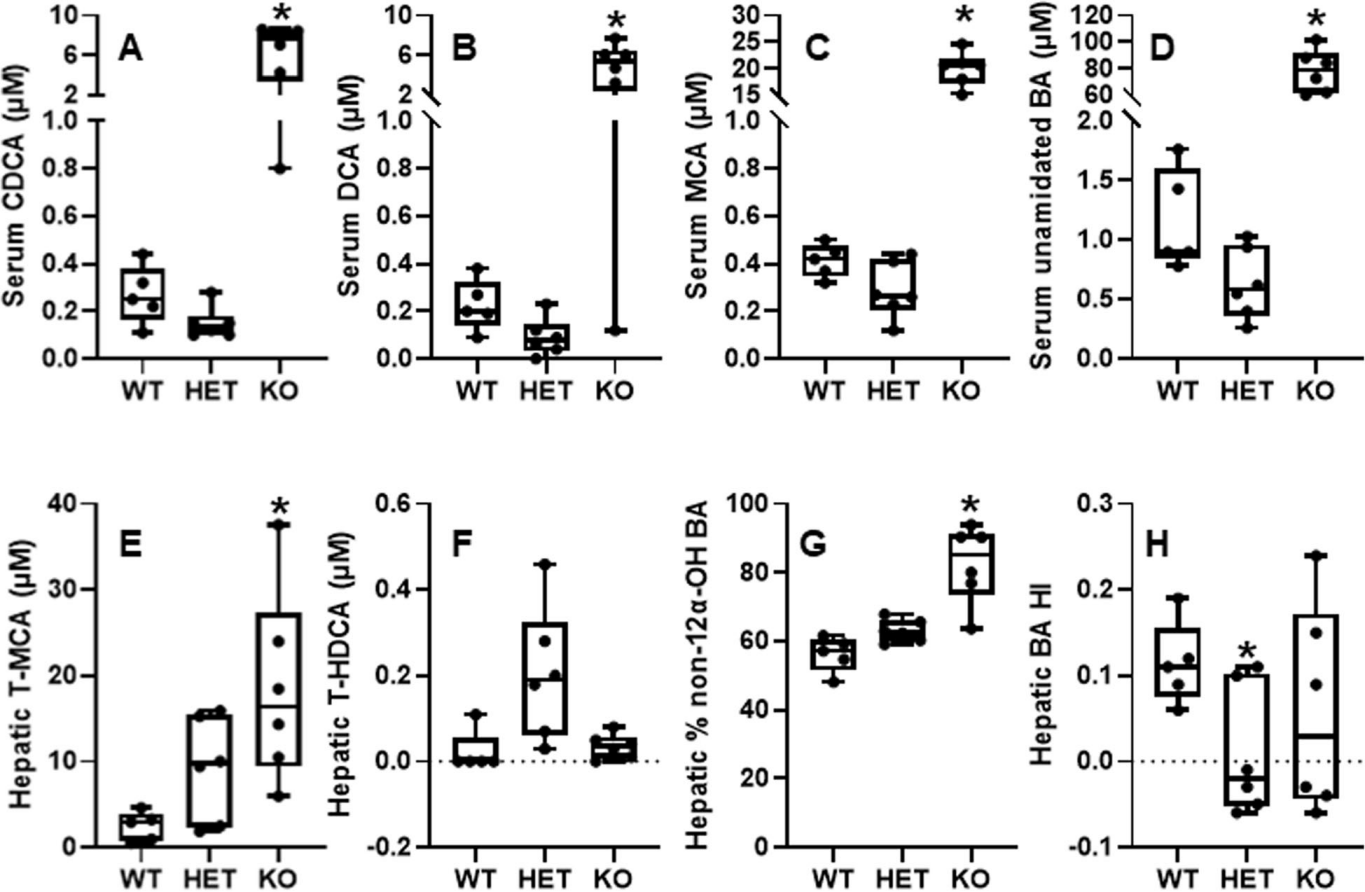
Serum and hepatic levels of bile acids in adult male wildtype (WT), HNF4α heterozygote (HET), and HNF4α knockout (KO) mice fed 15 d with high-fat-high-sugar diet (HFHS) (*N*=5-6 per group). Bile acids were quantified by LC-MS/MS. **A** serum levels of CDCA; **B** serum levels of DCA; **C** serum levels of MCA; **D** serum levels of unamidated bile acids; **E** hepatic levels of T-MCA; **F** hepatic levels of T-HDCA; **G** hepatic ratio of %non-12α-OH: total bile acids; **H** hepatic hydrophobicity index (HI) of bile acids. In the box plots, the end of the lower whisker is the minimum value, the boundary of the box closest to zero indicates the 25th percentile, a black line within the box marks the median, the boundary of the box farthest from zero indicates the 75th percentile, and the end of the upper whisker is the maximum value. * *P <* 0.05 versus WT mice

Interestingly, compared to WT mice, the HET mice had similar serum levels of total BAs, but tended to have less CDCA (↓54%, *p* = 0.066) (Fig. 4A), DCA (↓59%, *p* = 0.051) (Fig. 4B), MCA (↓30%, *p* = 0.062) (Fig. 4C), and total unamidated (unconjugated) BAs (↓45%, *p* = 0.053) (Fig. 4D), respectively. In contrast, the KO mice had significantly marked increases in CDCA (↑19.5 fold), DCA (↑19.5 fold), MCA (↑ 47.5 fold), and total unamidated BAs (↑77 fold). Additionally, the KO mice had marked increases in total amidated BAs (↑18 fold), unsulfated BAs (↑43 fold), sulfated BAs (↑30 fold), primary BAs (34 fold), secondary BAs (↑87 fold), mono-OH BAs (↑64 fold), Di-OH BAs (↑24 fold), Tri-OH BAs (↑36 fold), 12α-OH BAs (↑47 fold), and non-12α-OH BAs (↑40 fold), with a 42 fold increase in total BAs in the KO mice (Supplemental Figure 2, https://figshare.com/s/4a15e7089b4744443033). Moreover, certain sulfated (S) BAs, which were barely detectable in the WT mice, were significantly elevated in the KO mice, such as UDCA-S (36 nM), CDCA-S (25 nM), DCA-S (21 nM), CA-S (10 nM), and MCA-S (85 nM) (Supplemental Figure 2). Regarding the composition of total BAs, the HNF4α KO mice had marked 65%, 60%, and 58% decreases in % amidation of total BAs, % HCA, and % DCA respectively, whereas 1.1, 17, and 1.6 fold increases in % secondary BAs, % MDCA, and % UDCA, respectively. The overall hydrophobicity index (HI) of blood BAs remained unchanged in the blood of these mice.

##### Changes in hepatic bile acids in HFHS-fed HET mice

Compared to WT mice, most of the BAs remained unchanged in the HET mice. Interestingly, the HET mice had significant increases in T-MCA (↑2.8 fold) (Fig. 4E) and T-HDCA (↑8.4 fold) (Fig. 4F), without changes in hepatic total levels of MCA and HDCA (Supplemental Figure 2). The percentage of total non-12α-hydroxylated BAs significantly increased in the HET compared to WT mice (Fig. 4G), and the hydrophobicity index (HI) decreased in the HET compared to WT mice (Fig. 4H). The lower hydrophobicity index in the HET mice suggests a more hydrophilic BA pool, which may alter intestinal absorption of lipids [39].

##### Changes in hepatic bile acids in HFHS-fed KO mice

compared to HFHS-fed WT mice, the KO mice had a trend of decreased total CA (↓34%) but a marked 4.9 fold increase of total CDCA, which is associated with increases in free CDCA (↑4.5 fold), T-CDCA (↑6.0 fold), and oxo-CDCA (↑2.5 fold) (Supplemental Figure 2). Hepatic T-MCA markedly increased 6.6 fold, and free MCA also tended to increase (*p*=0.055) in the KO mice in which HNF4α was not knocked out until the adult age. The increases of MCA in the KO mice are consistent with the marked cholestasis and the known enterohepatic recycling of MCA.

Regarding secondary BAs, HDCA markedly decreased by 86%, whereas HCA increased 15 fold in the KO mice (Supplemental Figure 2), suggesting decreased bacterial conversion of LCA to HDCA but increased CYP3A-catalyzed conversion of CDCA to HCA in the KO mice. Total DCA tended to decrease in the KO mice, with a dramatic decrease of T-DCA (↓91%) and a trend of increase in free DCA. In contrast, hepatic levels of UDCA and LCA remained little changed (Supplemental Figure 2).

Sulfation of BAs plays an important role in the detoxification of BAs. T-CA sulfate (T-CA-S) and T-MCA sulfate (T-MCA-S), two major sulfated BAs in WT mouse livers, were dramatically decreased by 85 and 98%, respectively, in the KO mice (Supplemental Figure 2). Total sulfated BAs decreased by 97%, whereas the unsulfated BAs doubled in livers of the KO mice (Fig. 4D). Total amidated BAs tended to decrease (↓52%), whereas the total unamidated BAs increased 1.7 fold in the KO mice. Additionally, hepatic total levels of G-BAs, a minor form of amidated BAs in rodents, were 71% lower in the KO mice (Supplemental Figure 2).

#### Gene-dosage-distinct changes in hepatic transcriptome in HFHS-fed HNF4α HET and KO mice

**Fig. 5:**
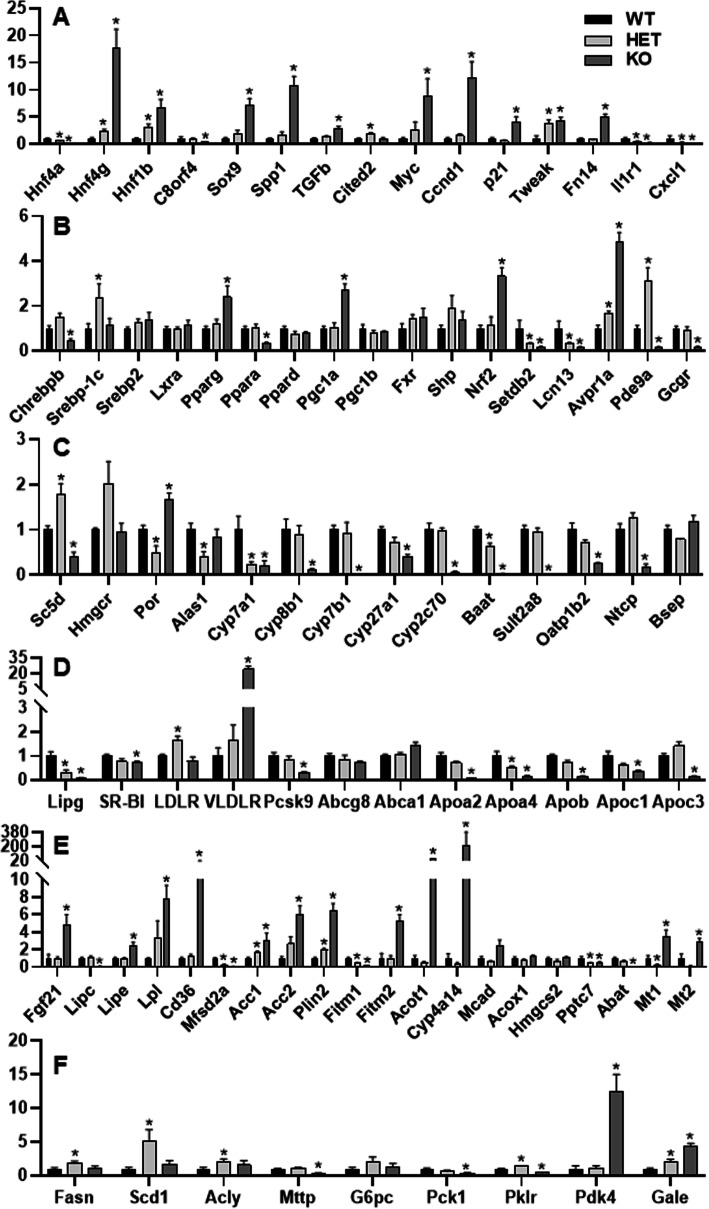
Real-time PCR quantification of hepatic mRNAs in adult male wildtype (WT), HNF4α heterozygote (HET), and HNF4α knockout (KO) mice fed the high-fat-high-sugar diet (HFHS) for 15 d. **A** genes important in the differentiation and proliferation of hepatocytes and cholangiocytes; **B** transcriptional regulators; **C** genes important for cholesterol and bile acid metabolism; **D** genes important for apolipoprotein metabolism; **E** genes important in lipid metabolism; and **F** genes important for the metabolism of fatty acids and sugar. *N*=4-6, mean ± SE. Data were normalized to peroxiredoxin 1 (Prxd1), with wildtype values set at 1.0. * *P <* 0.05 versus WT mice

**A. Gene-dosage-distinct changes in genes essential for hepatocyte proliferation and differentiation (Fig.** **5****A).** To understand the mechanism of changes in hepatic metabolism of lipids and bile acids, RNA-sequencing of pooled livers was conducted (Supplemental Table S3, https://figshare.com/s/cf6fab65ffa2384158bb) from all the mice in a given group (*N*=6-7 per group), followed by verification with real-time PCR using randomly-selected individual samples (*N*=4-6 per group) (Fig. 5). As expected, Hnf4a was gene-dosage-dependently decreased in the HNF4α HET and KO mice (Fig. 5A). HNF4α deficiency caused gene-dosage-dependent induction of HNF4g in HNF4α HET (↑1.3 fold) and KO (↑16.8 fold) mice, which might partially compensate for the loss of HNF4α. HNF4α is a well-established master regulator of hepatocyte differentiation and maturation, whereas HNF1β is critical for the differentiation of hepatoblasts into cholangiocytes [40]. A previous study found that Hnf1b was induced in HNF4α KO mice [12]. Interestingly, HNF1b was gene-dosage-dependently induced in HNF4α HET (↑2 fold) and KO (↑5.7 fold) mice. Hepatocytes and cholangiocytes have a common precursor of hepatoblasts. Cbp/p300-interacting transactivator 2 (Cited2), a co-activator of HNF4α, plays a key role in liver development [41]. Interestingly, cited2 was induced in HET, but not KO mice (Fig. 5A). The transcriptional and immune response regulator (TCIM/C8orf4) inhibits the self-renewal of liver cancer stem cells by suppressing NOTCH2 signaling [42]. The NOTCH2-SRY-box transcription factor 9 (SOX9) signaling promotes biliary epithelial cell specification during bile duct development and cholangiocarcinogenesis [43, 44]. Activation of Notch signaling in hepatocytes also promotes the secretion of osteopontin (SPP1), which promotes myofibroblast differentiation and liver fibrosis [44]. There was 71% down-regulation of C8orf4 in the KO mice and a clear trend of gene-dosage-dependent induction of Sox9, Spp1, and transforming growth factor beta1 (Tgfb1) in HNF4α HET (↑94%, 68%, and 25%, respectively) and KO (↑6.1, 9.8, and 1.9 fold, respectively) mice (Fig. 5A).

Total loss of HNF4α causes marked hepatocyte proliferation [29]. There was gene-dosage-dependent induction of c-Myc and cyclin d1 (Ccnd1) in HNF4α HET and KO mice (Fig. 5A). Induction of TNF-like weak inducer of apoptosis (TWEAK) ligand and its receptor fibroblast growth factor-inducible 14 (Fn14) plays a key role in hepatocyte proliferation during chronic liver diseases [45]. Interestingly, the TWEAK ligand was strongly induced in both the HET (↑2.8 fold) and KO (↑3.3 fold) mice, whereas the Fn14 receptor, which is not expressed in normal hepatocytes, was only induced in the KO mice (↑4.0 fold). Additionally, the CDK inhibitor p21, an HNF4α-target gene [46], tended to be down-regulated in the HET mice but strongly induced 3.0 fold in the KO mice (Fig. 5A). Taken together, these data clearly demonstrate a critical gene-dosage-dependent role of HNF4α in maintaining hepatocyte differentiation and suppressing hepatocyte proliferation, cholangiocyte transdifferentiation, and liver fibrosis during HFHS intake. The impaired differentiation and maturation of hepatocytes with the progressive loss of HNF4α will impede the hepatic metabolism of lipids and bile acids, a highly differentiated function of the liver.

Acute loss of HNF4α promotes rapid hepatocyte proliferation without the induction of inflammatory cytokines [29]; the underlying mechanism remains unknown. Chemokine (C-X-C motif) ligand 1 (CXCL1) and interleukin-1 receptor 1 (IL1R1) play key roles in regulating hepatic proinflammatory responses [47, 48]. HNF4α had gene-dosage-dependent critical roles in regulating the expression of Cxcl1 and Il1r1 in mouse livers: Cxcl1 was 79% and 95% lower, whereas Il1r1 was 60% and 81% lower in the HET and KO mice (Fig. 5A). Hepatic down-regulation of Cxcl1 and Il1r1 may play important roles in preventing the infiltration of inflammatory cells and activation of the inflammatory kinase pathways in the HNF4α KO mice [29] (Fig. 3).

**B. Gene-dosage-distinct changes in transcriptional regulators** (**Fig.** **5****B**)**.** Carbohydrate-responsive element-binding protein (ChREBP), SREBP-1, and LXRs are key lipogenic transcription factors. HNF4α is essential for the transactivation of ChREBPb, a constitutive-active isoform of ChREBP [49]. HNF4α KO mice had 53% lower Chrebpb than WT mice (Fig. 5B). In contrast, HNF4α HET mice had 1.4 fold higher Srebp-1c and a trend of higher (48%) Chrebpb than WT mice. PPAR family members regulate genes in lipid and carbohydrate metabolism. Hepatic mRNA expression of Srebp2, Lxrα, and Ppard remained unchanged in these mice (Fig. 5B). In contrast, Ppara was down-regulated 65%, whereas Pparg was up-regulated 1.4 fold in the KO mice. PPARγ coactivator 1-alpha (PGC1α) is a master regulator of mitochondrial biogenesis and gluconeogenesis. PGC1a, but not PGC1b, was induced 1.7 fold in the KO mice. The BA receptor farnesoid X receptor (FXR) and its target gene orphan nuclear receptor SHP play key roles in BA and lipid metabolism [50]. SHP is induced in NAFLD, and SHP induction promotes steatosis but inhibits inflammation, partly via induction of PPARγ and suppression of NF-kB [51]. FXR and SHP tended to be higher in the HET and KO mice. Consistent with a previous report on the induction of antioxidative genes [12], NF-E2-related factor-2 (Nrf2), a master regulator of antioxidative responses, was induced 2.3 fold in the KO mice (Fig. 5B). Induction of the epigenetic modifier SET domain bifurcated histone lysine methyltransferase 2 (SetDB2) by GR ameliorates fatty liver [25]. Lipocalin 13 (LCN13) protects against fatty liver by inhibiting lipogenesis and stimulating fatty acid (FA) β-oxidation (FAO) [52]. Plasma vasopressin (VP) is increased in diabetic patients and promotes fatty liver by activating hepatic arginine VP receptor 1A (Avpr1a) [53, 54]. Hepatic expression of Setdb2, Lcn13, and Avpr1a were highly gene-dosage-dependent on HNF4α (Fig. 5B): Setdb2 was 69% and 82% lower, Lcn13 was 69% and 86% lower, whereas Avpr1a was 69% and 3.8 fold higher, in the HET and KO mice than WT mice, respectively. Loss of hepatic glucagon receptor (GCGR) lowers blood glucose and increases insulin sensitivity [55]. Inhibition of the cGMP-specific phosphodiesterase 9A (PDE9A) decreases gluconeogenesis and blood glucose levels [56]. Hepatic Gcgr and Pde9a were markedly down-regulated in KO mice but unchanged or up-regulated in the HET mice (Fig. 5B), which may contribute to the decreased blood glucose levels in the KO mice (Table 1).

**C. Gene-dosage-dependent changes of genes important in cholesterol and BA metabolism** (**Fig.** **5****C & D**)**.**

Lathosterol 5-desaturase (Sc5d), essential for cholesterol synthesis [57], was 79% higher in HET but 59% lower in KO mice than WT mice (Fig. 5C). HMG-CoA reductase (HMGCR), the rate-limiting enzyme for cholesterol biosynthesis, was induced 1.0 fold in the HET but remained unchanged in the KO mice. Cytochrome P450 reductase (POR) is essential in lipid metabolism by maintaining the activities of all P450s [58], whereas the liver-predominant aminolevulinic acid synthase 1 (ALAS1) is rate-limiting in heme synthesis whose deficiency contributes to mitochondrial dysfunction and NAFLD progression. Interestingly, Por and Alas1 mRNAs were 51% and 59% lower, respectively, in HET mice than in WT mice (Fig. 5C). Importantly, Por HET mice have a ~50% decrease in Por activity [59], suggesting decreased POR activity in HET mice.

CYP7A1 is important in cholesterol catabolism and the prevention of HFD-induced fatty liver [60]. Cyp7a1 was down-regulated in both HET (↓78%) and KO (↓80%) mice, whereas only the KO mice had significant down-regulation of Cyp8b1 (↓91%), Cyp7b1 (↓97%), Cyp27a1 (↓59%), and Cyp2c70 (↓94%) (Fig. 5C). Consistent with a previous report [61], bile acid-CoA: amino acid N-acyltransferase (BAAT), the key enzyme for amino acid conjugation of bile acids, was down-regulated 98% in the KO mice. Sulfotransferase 2a8 (Sult2a8), a newly identified major hepatic BA sulfonating enzyme in mice [62], was dramatically down-regulated (↓99%) in the KO mice. Consistent with a previous report [12], expression of Na+-taurocholate cotransporting polypeptide (NTCP) and organic anion transporting polypeptide 1b2 (Oatp1b2), key uptake transporters for conjugated and unconjugated BAs [63], were markedly down-regulated 82% and 75%, respectively, in the KO mice. In contrast, hepatic expression of the bile salt export pump (BSEP) remained unchanged in the KO mice (Fig. 5C).

Hepatic endothelial lipase (EL/LIPG) reduces blood levels of HDL cholesterol by promoting HDL catabolism and hepatic uptake of HDL cholesterol [64]. Lipg was gene-dosage-dependently down-regulated in the HET (↓68%) and KO (↓92%) mice (Fig. 5D). Scavenger receptor class B, type I (SR-BI), LDL receptor (LDLR), and VLDL receptor (VLDLR) are essential for hepatic uptake of HDL, LDL, and VLDL cholesterol, respectively. Ldlr was 64% higher, and Vldlr also tended to be higher in the HET mice. In contrast, SR-BI was slightly decreased by 27%, whereas Vldlr, a PPARα/PPARγ-target gene [65], was remarkably induced 23-fold in the KO mice. Although Ldlr mRNA was not significantly altered in the KO mice, proprotein convertase subtilisin/kexin type 9 (PCSK9), which plays a key role in the degradation of LDLR protein, was markedly decreased by 70%, suggesting that hepatic protein levels of LDLR might be elevated in the KO mice. Thus, the HET and KO mice likely have decreased hepatic uptake of HDL but increased uptake of VLDL/LDL cholesterol. Additionally, hepatic expression of the biliary cholesterol efflux transporter Abcg8 remained unchanged, whereas the basolateral cholesterol efflux transporter ATP binding cassette subfamily A member 1 (Abca1) tended to be higher in the KO mice (Fig. 5D). The apolipoproteins Apo-AI and Apo-AII have major roles in regulating HDL cholesterol and lipid metabolism [66]. Chow-fed mice with deficiency of Apolipoprotein a2 (Apoa2) have 64% and 32% decreases in blood levels of HDL cholesterol and free fatty acids, without changes in non-HDL cholesterol and triglycerides [67]. Consistent with the previous report of chow-fed HNF4α KO mice [13], HFHS-fed HNF4α KO mice had dramatic down-regulation of Apoa2 (↓94%), Apoa4 (↓85%), Apob (↓86%), Apoc1 (↓65%), and Apoc3 (↓85%) (Fig. 5D), but little changes in Apoa1 and Apoe (data not shown).

**D. Gene-dosage-dependent changes of genes important in lipid metabolism (Fig.** **5****E).**

Fibroblast growth factor 21 (FGF21) is a hepatokine that increases insulin sensitivity and regulates lipolysis in white adipose tissue [68]. Hepatic Fgf21 was 3.8 fold higher in the KO mice (Fig. 5E). The liver-predominant Lipase C, Hepatic Type (LIPC) and fat-predominant lipoprotein lipase (LPL) mediate lipoprotein hydrolysis to provide free fatty acids for energy and storage. Loss of Lipc protects against, whereas liver-specific overexpression of LPL promotes diet-induced obesity and hepatic steatosis [69, 70]. In contrast, hepatic overexpression of the lipase E, hormone sensitive type (HSL/LIPE) ameliorates fatty liver by promoting lipolysis and fatty acid oxidation [71]. Interestingly, Lipc and Lipe were 94% lower and 1.4 fold higher in the KO mice, which may help ameliorate fatty liver in these mice. In contrast, Lpl tended to be gene-dosage-dependently increased in the HET (↑2.3 fold) and KO (↑6.8 fold) mice (Fig. 5E). The free fatty acids released by these lipases are taken up in the liver by fatty acid translocase (FAT/CD36). Hepatocyte-specific disruption of CD36 attenuates fatty liver and improves insulin sensitivity in HFD-fed mice, with large decreases in hepatic oleic acid without change in the essential ω3 FAs [72]. In contrast, major facilitator super family domain containing 2a (MFSD2A) is a key transporter for ω3 FAs which is essential for the prevention of fatty liver [73]. Cd36 was strongly induced 20 fold in the KO mice, whereas Mfsd2a was 77% and 94% lower in the HET and KO mice than WT mice, respectively, which may result in a marked increase in hepatic uptake of oleic acid in the KO mice but decreases in the uptake of ω3 FAs in the HET and KO mice.

The FAs taken up into the liver will be utilized or stored. The present study found that the HET and KO mice had gene-dosage-dependent induction of key genes that inhibit lipid catabolism (Fig. 5E). Acetyl-CoA carboxylase (ACC) Acc1 and Acc2 are key lipogenic genes by inhibiting β-oxidation and promoting *de novo* lipogenesis. Perilipin 2 (PLIN2), a lipid droplet protein highly up-regulated in steatotic livers [74], promotes fatty liver and fibrosis [75]. HNF4α had a gene-dosage-dependent critical role in preventing hepatic up-regulation of Acc1, Acc2, and Plin2 (Fig. 5E): Acc1 was 65% and 2.0 fold higher, Acc2 was 1.6 and 5.0 fold higher, whereas Plin2 was 98% and 5.5 fold higher, in HFHS-fed HNF4α HET and KO mice than WT mice, respectively. The muscle-predominant fat storage-inducing transmembrane protein 1 (FITM1) promotes the formation of smaller lipid droplets, likely to turn over FAs for mitochondrial β-oxidation [76]. In contrast, overexpression of FITM2 increases lipid droplets and triglycerides in mouse liver [77]. Interestingly, Fitm1 was 56% and 90% lower in HET and KO mice than WT mice, respectively, whereas Fitm2 was 4.2 fold higher in the KO mice. FAs must be converted to acyl-CoAs for mitochondrial FAO. Acyl-CoA thioesterase 1 (ACOT1) hydrolyzes acyl-CoAs back to CoA and free FAs which promote fatty liver but inhibit inflammation and oxidative stress by activating PPARα [78]. Additionally, the PPARα-target genes, namely the microsomal Cyp4a14, the peroxisomal acyl-coenzyme A oxidase 1 (ACOX1), as well as the mitochondrial enzymes medium-chain acyl-CoA dehydrogenase (MCAD), and hydroxymethylglutaryl-CoA synthase, mitochondrial (HMGCS2) play key roles in FAO [27]. All these PPARα-target genes tended to be lower in the HET mice, whereas Cyp4a14 was markedly induced (↑213 fold) and MCAD tended to be induced (↑1.5 fold, *p* = 0.056) in the KO mice (Fig. 5E). The trend of induction of MCAD may be due to the induction of PGC1a (Fig. 5B) which can strongly induce MCAD independent of HNF4α [79]. Protein Phosphatase Targeting COQ7 (Pptc7) is a newly identified essential phosphatase for promoting mitochondrial metabolism and biogenesis [80]. Interestingly, Pptc7 was down-regulated in both the HET (↓56%) and KO (↓49%) mice (Fig. 5E). The gut-derived γ-Aminobutyric Acid (GABA) is accumulated in liver disease and contributes to hepatic encephalopathy in patients with cirrhosis [81]. 4-aminobutyrate aminotransferase (ABAT), a key enzyme for GABA catabolism and mitochondrial nucleoside metabolism [82], tended to be gene-dosage-dependently decreased in the HET and KO mice (↓94%). Interestingly, GABA potently protects against severe liver injury [83]. Thus, the dramatic down-regulation of ABAT in the KO mice may impair mitochondrial nucleoside metabolism but protect the KO mice from liver injury via elevated GABA. Metallothionein protects against HFD-induced fatty liver [84]. Metallothionein-1 (Mt1) and Mt2 are the most markedly down-regulated genes in the HFD-fed obesity-prone C57/BL6 mice [85]. Mt1 and Mt2 were markedly decreased by 79% and 91%, respectively, in HET mice but increased 2.5- and 1.9-fold in KO mice (Fig. 5E), which may be due to stress responses such as activation of Nrf2 and FXR by cholestatic liver injury in these mice [12, 86].

**E. Differential alterations of key genes for lipogenesis and sugar metabolism (Fig.** **5****F)**.

HNF4α HET mice had induction of key lipogenic enzymes FA synthase (Fasn) (↑79%) and stearoyl-CoA desaturase-1 (Scd1) (↑4.2 fold). ATP citrate lyase (ACLY), a sterol regulatory element binding transcription factor 1 (Srebp-1)-target key enzyme in *de novo* FA and cholesterol synthesis [87], was 1.1 fold and 56% higher in the HET and KO mice. Additionally, KO mice had 68% down-regulation of microsomal triglyceride transfer protein (Mttp) (Fig. 5F).

The metabolism of sugar and lipids is closely interrelated. Liver-type pyruvate kinase (PKLR), essential in glycolysis and lipogenesis [88], was 40% higher in HET but 63% lower in KO mice (Fig. 5F). Hepatic Pyruvate dehydrogenase kinase 4 (PDK4) levels are highly induced in human patients with NASH, whereas deletion of Pdk4 improves fatty liver in mice [89]. Pdk4 was markedly induced 11.5 fold in the KO mice. Interestingly, HNF4α deficiency in HFHS-fed mice had distinct effects on phosphoenolpyruvate carboxykinase 1 (PEPCK/PCK1) and glucose-6-phosphatase catalytic subunit (G6PC), two key enzymes for gluconeogenesis. Pepck tended to be gene-dosage-dependently down-regulated (↓65% in KO mice), whereas G6pc tended to be up-regulated in HET and KO mice. Pepck promotes gluconeogenesis but protects against fatty liver [90]. The marked down-regulation of Pepck likely contributes to decreased blood glucose and accumulation of lipids in the HFHS-fed KO mice. Hepatic expression of UDP-galactose-4-epimerase (GALE), the last enzyme in the conversion of UDP-galactose to UDP-glucose, is induced after long-term HFHS feeding [91]. Hepatic induction of GALE impairs insulin sensitivity [91]. Interestingly, Gale was gene-dosage-dependently induced in the HET (↑1.1 fold) and KO (↑ 3.3 fold) mice (Fig. 5F), suggesting a key role of HNF4α in preventing hepatic induction of GALE during HFHS intake.

#### Western blotting quantification of protein expression of genes essential for lipid metabolism in HNF4α HET and KO mice fed HFHS for 15 d

**Fig. 6:**
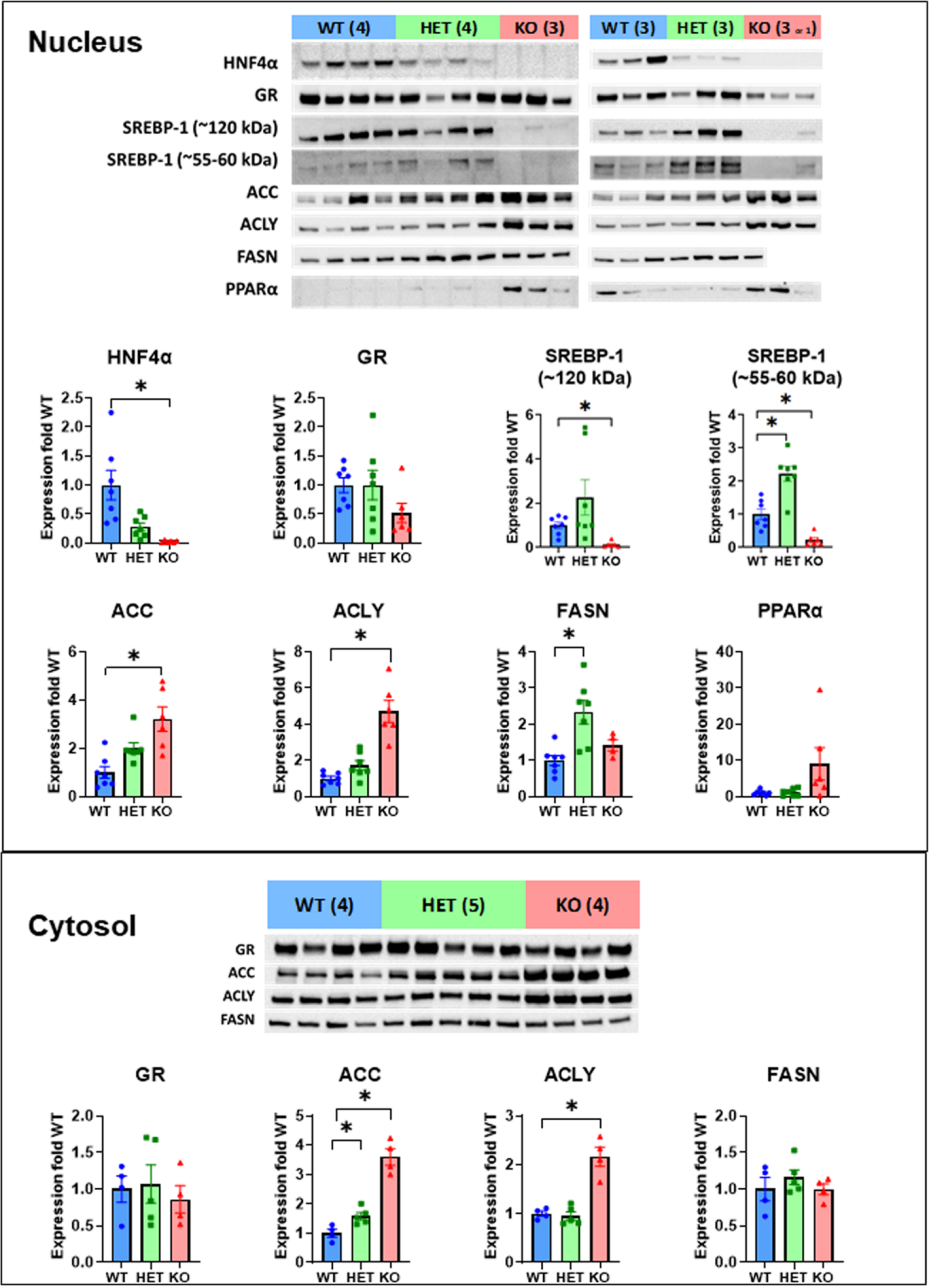
Western blotting quantification of proteins in hepatic nuclear (top) and cytosolic (bottom) extracts in adult male wildtype (WT), HNF4α heterozygote (HET), and HNF4α knockout (KO) mice fed the high-fat-high-sugar diet for 15 d. *N*=4-7 per group, Mean ± SE. * *P <* 0.05 versus WT mice

HNF4α HET and KO mice had expected partial (↓72%) and complete (↓98%) loss of HNF4α proteins (Fig. 6). The SREBP-1 protein is synthesized as a precursor form (~120 kDa) anchored in the endoplasmic reticulum and nuclear membranes. After proteolytic cleavage, the mature active form (~55-60 kDa) of SREBP-1c migrates into the nucleus [92]. HNF4α HET mice had a trend of increase (↑1.3 fold) in nuclear precursor form (120 KD) (Fig. 6) and a significant increase (↑1.2 fold) in the mature form of SREBP-1C proteins (55-60 KD) (Fig. 6). In contrast, both the nuclear precursor and mature forms of SREBP-1 proteins were markedly decreased in HNF4α KO mice (↓88% and 77% respectively). Consistent with the gene-dosage-dependent induction of Acc mRNAs (Fig. 5E), nuclear ACC proteins tended to increase moderately (↑1.0 fold), whereas the cytosolic ACC proteins significantly increased (↑60%) in HNF4α HET mice. Both the nuclear (↑ 2.2 fold) and cytosolic (↑2.6 fold) ACC proteins were markedly increased in HNF4α KO mice (Fig. 6). In contrast to more marked increases of Acly mRNAs in the HNF4α HET than in the HNF4α KO mice (Fig. 5F), ACLY proteins were only significantly increased in the nucleus (↑3.7 fold) and cytosol (↑1.2 fold) of HNF4α KO mice (Fig. 6). Consistent with changes in Fasn mRNAs, FASN proteins increased in the nucleus in the HNF4α HET mice (↑1.3 fold) and remained unchanged in the KO mice, although the cytosolic FASN proteins were unchanged in all the mice (Fig. 6). Surprisingly, nuclear PPARα proteins tended to be increased in the HNF4α KO mice (↑9.1 fold average, range 0.2 to 29.5) (Fig. 6), despite moderately decreased Ppara mRNAs in these mice (Fig. 5B). PPARα is a short-lived protein stabilized by its ligands [93]. Thus, the trend of increased PPARα proteins in the HNF4α KO mice might be due to ligand stabilization. 11β-hydroxysteroid dehydrogenase type 1 (HSD11B1) is responsible for the local activation of GR [94]. Hepatic Gr and Hsd11b1 mRNAs were unchanged in HNF4α HET and KO mice (data not shown). Nevertheless, nuclear GR proteins tended to be decreased in HNF4α KO mice (↓48%), whereas cytosolic GR proteins were unchanged in these mice (Fig. 6). CDCA selectively inhibits HSD11B1 activity (IC_50_ 2.8 μM) [95]. Thus, marked increases in serum and hepatic CDCA (Fig. 4) likely cause an inhibition of HSD11B1 and the resultant decreased nuclear levels of GR proteins in the HNF4α KO mice.

#### Suppression of mouse and human SREBP-1C promoter by HNF4α

**Fig. 7:**
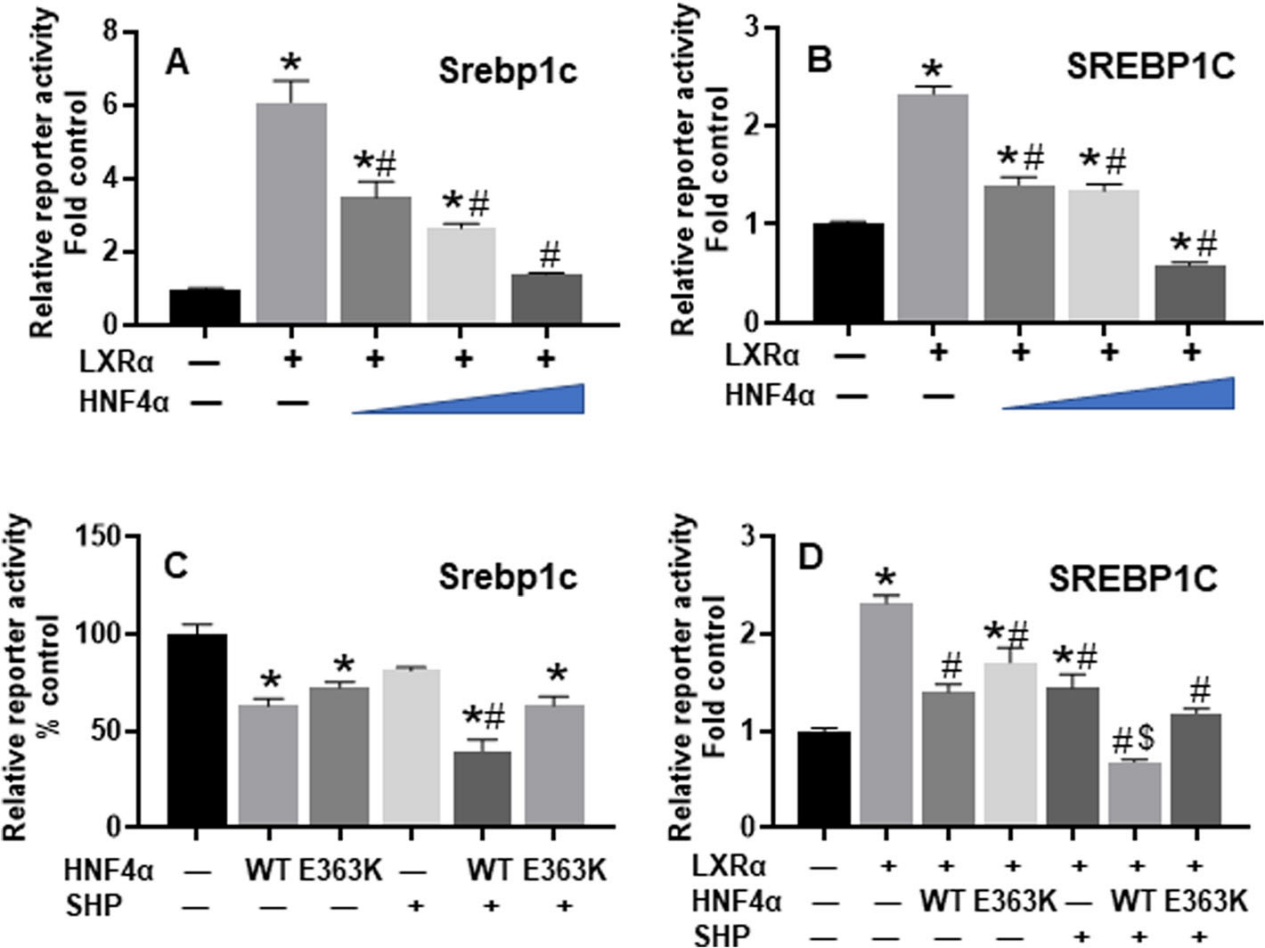
Dual-luciferase assays of regulation of mouse Srebp-1c and human SREBP-1C promoters. Assays were conducted 24 h after HEK293 cells (**A** - **C**) or HepG2 cells (**D**) were transfected with firefly reporter vectors, pRL-CMV, and expression vectors for HNF4α, LXRα, or SHP. *N*=4 per group, mean ± SE. * *P <* 0.05 versus control; # *P <* 0.05 versus LXRα (**A** & **B**), wildtype (WT) HNF4α (**C**), or LXRα (**D**) alone; $ *P <* 0.05 versus LXRα + SHP group

SREBP-1C and LXR are two master regulators of lipogenesis. The basal expression of SREBP-1C is dependent on LXR, a key lipogenic nuclear receptor that is activated by oxysterol [28]. Without a DNA-binding domain, the orphan receptor SHP mainly acts as a co-repressor [96]. Bile acids lower hepatic TG via the FXR-SHP-LXR-SREBP-1c pathway [97]. The present study found that HNF4α gene-dosage-dependently suppressed the LXR-mediated transactivation of reporters for the mouse (Fig. 7A) and human SREBP-1C (Fig. 7B) promoter in HEK293 cells. An E363K mutation in the AF-2 activation domain of HNF4α does not affect its cellular protein expression [98] but blocks its interaction with SHP [99]. In hepatoma cells, SHP potently inhibits the transactivation of Srebp-1c by LXRα [28]. In HEK293 cells that lack HNF4α expression, this study found that SHP did not significantly inhibit the basal activity of the Srebp-1c promoter (Fig. 7C). Both the WT and E363K mutant HNF4α moderately inhibited the mouse Srebp-1c promoter in HEK293 cells, whereas WT HNF4α, but not the SHP-interaction-defective E363K mutant HNF4α, cooperated with SHP to synergistically inhibit Srebp-1c promoter activities (Fig. 7C). Moreover, WT HNF4α, but not the SHP-interaction-defective E363K mutant HNF4α, cooperated with SHP to synergistically inhibit the LXRα-transactivation of human SREBP-1C promoter in HepG2 cells (Fig. 7D). Thus, SHP mediates the strong inhibition of LXR-transactivation of human SREBP-1C and mouse Srebp-1c by HNF4α.

#### Crosstalk of GR with HNF4α and PPARα in the regulation of hepatic gene expression

**Fig. 8:**
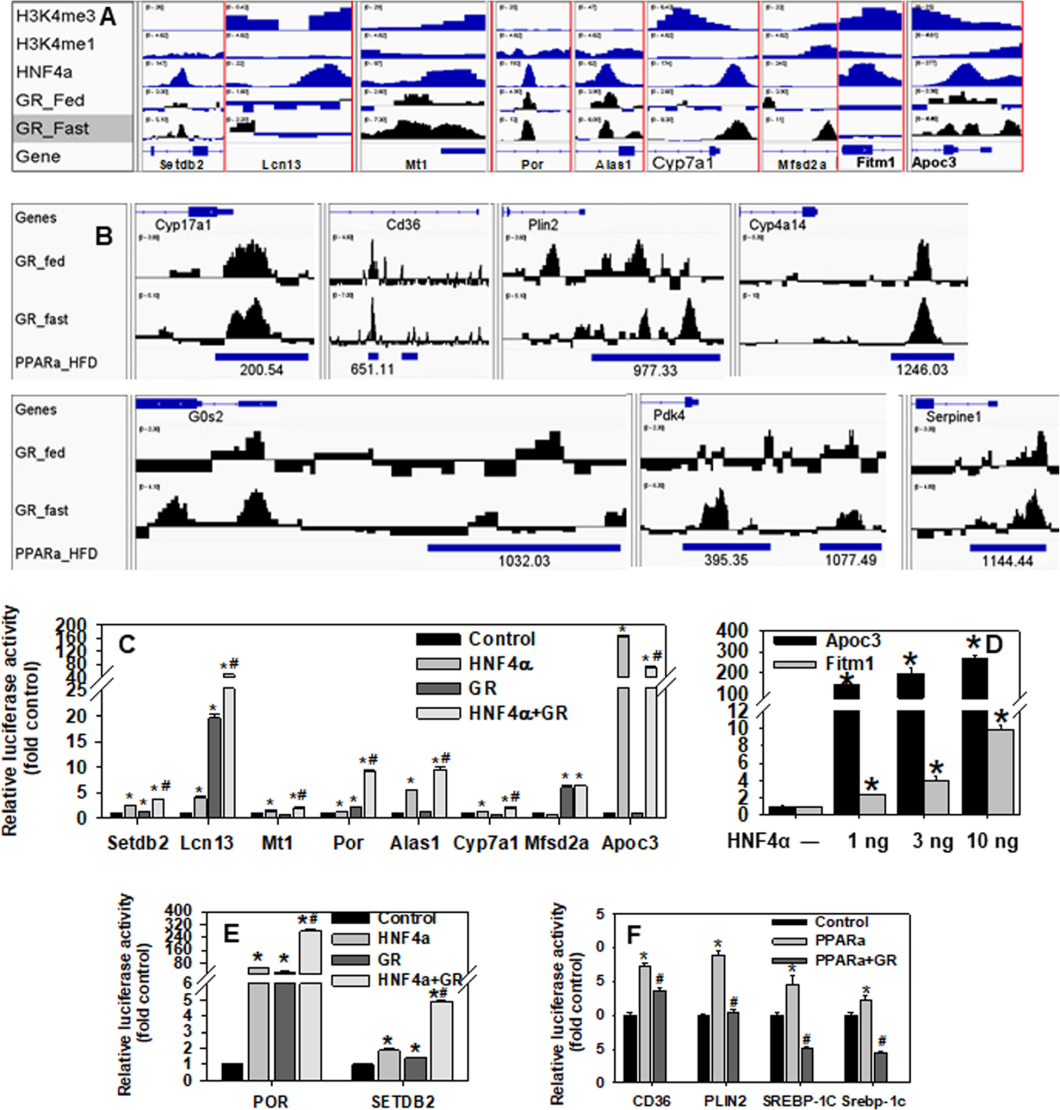
Interactions of GR with HNF4α and PPARα in gene regulation. **A** Analysis of ChIP-sequencing data of DNA-binding of epigenetic signatures, HNF4α, and GR in gene loci in wildtype mouse liver. **B** Analysis of ChIP-sequencing data of DNA-binding of GR and PPARα in gene loci in wildtype mouse liver. **C** - **F** Dual-luciferase assays of promoter/intron activities of mouse genes (**C**-**D**), human POR and SETDB2 (**E**), as well as human CD36, human PLIN2, human SREBP-1C, and mouse Srebp-1c (**F**) genes in HEK293 cells 24 h after transfection with firefly reporter vectors, pRL-CMV, and expression vectors for HNF4α, GR (with 10 nM dexamethasone), or PPARα (with 10 μM clofibrate). *N*=4 per group, mean ± SE. * *P <* 0.05 versus control; # *P <* 0.05 versus HNF4α or PPARα alone

GR and HNF4α can cooperate to induce CYP2A6 and PEPCK in humans [100, 101]. Additionally, GR can gene-specifically enhance or antagonize the transactivation by PPARα [102, 103], a master regulator of lipid metabolism. Thus, this study hypothesized that down-regulation of GR signaling (Fig. 6) plays a key role in the dysregulation of HNF4α- and PPARα-target genes in HNF4α HET and/or KO mice. Histone H3 trimethylation at lysine-4 (H3K4me3) is a marker for active transcription, whereas H3K4me1 is a marker for active enhancers [104]. The public datasets of chromatin immunoprecipitation (ChIP)-sequencing of H3K4me3 (GSM769014), H3K4me1 (GSM722760), HNF4α (GSM1390711), GR in the fed state (GR_Fed, GSM1936962), and GR in the 24h-fasted state (GR_Fast, GSM1936964) in WT mouse livers were uploaded into the Integrative Genomics Viewer (IGV) software [105] to visualize their DNA-binding in each gene locus. Both HNF4α peaks and enhanced GR-binding after fasting were found in promoters of Lcn13, Cyp7a1, Mt1, and Apoc3 as well as intron5 of Setdb2, intron1 of Por, and intron9 of Alas1, except that only HNF4α was found in Fitm1 exon1 and GR was found in Mfsd2a intron2 (Fig. 8A).

GR and PPARα can antagonize each other. IGV was used to visualize the DNA-binding of GR (in fed and fasted states) and PPARα (in HFD-fed state, GSE47954). Binding of GR and PPARα largely overlapped in the promoters/introns of Cyp17a1, Cd36, Plin2, Cyp4a14, G0/G1 switch gene 2 (G0s2), Pdk4, and plasminogen activator inhibitor-1 (PAI-1/Serpine1) (Fig. 8B).

Reporter vectors were generated by PCR-cloning the DNA elements in genes that show strong binding of GR and/or HNF4α in the ChIP-sequencing data (Fig. 8A), into the pGL3-Basic vector. Dual-luciferase assays were conducted 24 h after the HEK293 cells were co-transfected with the reporter vectors and expression vectors of HNF4α and/or GR (with 10 nM dexamethasone). HNF4α and GR synergistically/additively transactivated Setdb2, Lcn13, Mt1, Por, Alas1, and Cyp7a1 (Fig. 8C), which explains the down-regulation of these genes in HNF4α HET mice. In contrast, only GR transactivated Mfsd2a, whereas GR antagonized the strong transactivation of Apoc3 by HNF4α (Fig. 8C). Moreover, HNF4α and GR also synergistically transactivated human POR and SETDB2 introns (Fig. 8E). Additionally, HNF4α highly gene-dosage-dependently transactivated Fitm1 promoter, whereas the Apoc3 promoter was very strongly transactivated by merely 1 ng of HNF4α expression vector (Fig. 8D). Thus, the dramatic down-regulation of Apoc3 in HNF4α KO mice (Fig. 5D) may be due to the loss of HNF4α as an obligatory transactivator, whereas the trend of induction of Apoc3 in HET mice might be due to the decreased suppressing effects of GR on Apoc3.

A previous study demonstrates that certain PPARα-target genes are highly induced in chow-fed HNF4α KO mice despite hepatic down-regulation of PPARα [13]. PPARα induces the key lipogenic transcription factor Srebp-1c by directly binding to its promoter [106]. GR completely blocked the transactivation of human SREBP-1C and mouse Srebp-1c promoters by PPARα (Fig. 8F). Activation of PPARα induces key lipogenic genes PLIN2 and CD36 in mouse and human hepatocytes [107–109]. GR completely and partially blocked*,* respectively*,* the activation of intron1 of human PLIN2 and CD36 by PPARα (Fig. 8F).

#### Hepatic lipid accumulation and gene dysregulation in GR KO mice fed HFHS for 15 d

**Fig. 9:**
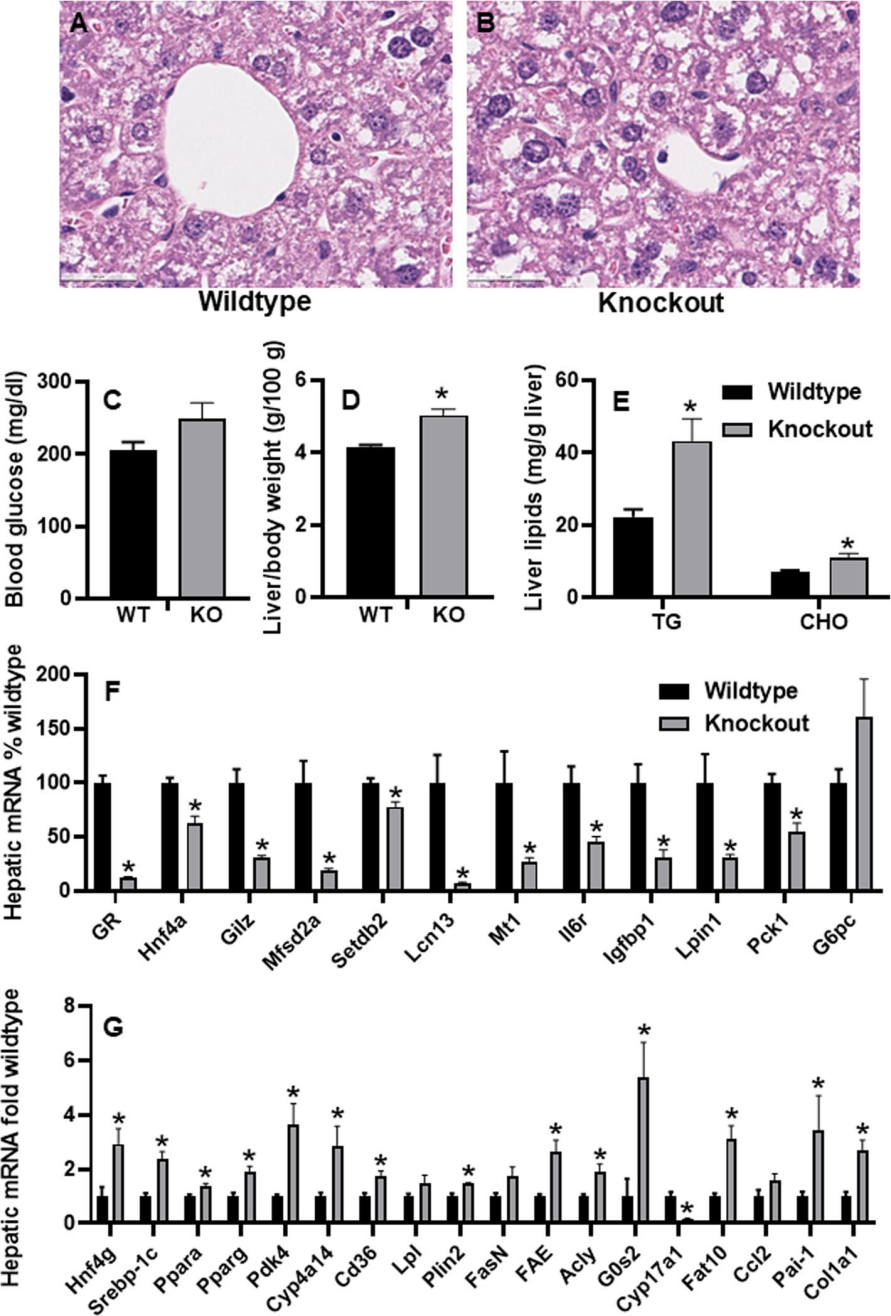
Effects of liver-specific knockout (KO) of glucocorticoid receptor (GR) on hepatic mRNA expression and lipid metabolism in high-fat-high-sugar (HFHS)-fed adult male mice. **A** & **B** Liver histology (H & E staining, 5 μm, 400X); **C** blood glucose; **D** liver/body weight ratio; **E** hepatic lipids, and **F** & **G** real-time PCR quantification of hepatic mRNAs. *N* = 6-7 per group, mean ± SE. Hepatic mRNAs were normalized to Pgk1, with wildtype values set at 1.0 or 100%. * *P <* 0.05 versus wildtype (WT) control

To definitively determine the role of hepatic GR in HFHS-induced lipid disorders, hepatic GR was specifically deleted in adult male mice and these mice were fed with HFHS for 15 d. Histology showed that GR KO mice (Fig. 9B) had more hepatocyte vacuolization than the WT mice (Fig. 9A). Blood glucose tended to be higher in GR-KO than WT mice (Fig. 9C). The liver/body weight ratio was 21% higher in GR KO mice than in WT mice (Fig. 9D), and hepatic levels of triglycerides and cholesterol in GR KO mice were 95% and 56% higher than WT mice (Fig. 9E), respectively. Blood levels of triglycerides and cholesterol were similar between WT and GR KO mice (data not shown). To understand the mechanism of aggravated lipid accumulation in HFHS-fed GR KO mice, real-time PCR was used to determine changes in hepatic mRNA expression. As expected, hepatic expression of exon 3 of GR mRNA was remarkably decreased (↓88%), demonstrating the high efficiency of hepatic GR deletion in these inducible GR KO mice (Fig. 9F). GR induces HNF4α in primary human hepatocytes [110]. GR KO mice had marked down-regulation of GR-target genes, namely Hnf4a (↓37%), glucocorticoid-induced leucine zipper (Gilz, ↓69%), Mfsd2a (↓80%), Setdb2 (↓22%), Lcn13 (↓92%), Mt1 (↓71%), IL6 receptor (Il6ra, ↓54%) [111], insulin like growth factor binding protein 1 (Igfbp1, ↓69%) [112], Lipin 1 (Lpin1, ↓70%) [113], and Pck1 (↓45%) (Fig. 9F). Low circulating IGFBP1 is associated with insulin resistance, diabetes, and cardiovascular disease [114]. Lpin1 activates PPARα to induce genes important for fatty acid oxidation [113]. Liver-specific lpin1 deficiency exacerbates experimental alcohol-induced steatohepatitis in mice [115]. In line with a trend of higher blood glucose, hepatic G6pc tended to be higher (↑61%) in GR KO mice (Fig. 9F). In this regard, high glucose induces glucotoxicity and G6pc expression via activating hypoxia-inducible factor (HIF)-1α or the CREB-binding protein [116].

In contrast to the down-regulation of HNF4α, HNF4γ was strongly induced (↑1.9 fold) in GR KO mice. Consistent with increased hepatic lipid accumulation, GR KO mice had induction of Pparα (↑37%) and key lipogenic transcription factors Pparg (↑92%) and Srebp-1c (↑1.4 fold), lipogenic genes Pdk4 (↑2.7 fold), Plin2 (↑46%), fatty acid synthase (Fasn, ↑74%), long-chain fatty acid elongase (FAE/Elovl6, ↑1.6 fold), Acly (↑91%), G0s2 (↑4.4 fold), and Cd36 (↑72%), as well as the trend of induction of Lpl (↑50%, *p* = 0.07) (Fig. 9G). G0S2 is a potent inhibitor of lipolysis and lipid droplet degradation [117]. ACLY is a critical enzyme linking glucose catabolism to lipogenesis [118]. Hepatic induction of these lipogenic genes may contribute to the aggravated fatty liver in these GR KO mice. In the liver, the classical steroidogenic enzyme Cyp17a1 catalyzes the production of at least one hormone-ligand, dehydroepiandrosterone, for the nuclear receptor PPARα [119]. Hepatic Cyp17a1 mRNA was markedly decreased by 83% in the GR KO mice. Additionally, GR KO mice tended to have higher proinflammatory cytokine C-C motif chemokine ligand 2 (Ccl2) (↑57%, *p* = 0.09). HLA-F-adjacent transcript 10 (FAT10) is an immune-cell-enriched ubiquitin-like modifier dramatically induced in liver injury and chronic liver diseases [120]. GR KO mice had 2.1 fold higher Fat10 than WT mice. PAI-1 plays a key role in steatosis, inflammation, and fibrosis [121–123]. GR KO mice had 2.4-fold higher Pai-1 and 1.7-fold higher collagen 1a1 (Col1a1) (Fig. 9G) mRNAs, suggesting a role of hepatocellular GR in the protection against HFHS-induced liver fibrosis.

#### Changes in hepatic protein levels in GR KO mice fed HFHS for 15 d

**Fig. 10:**
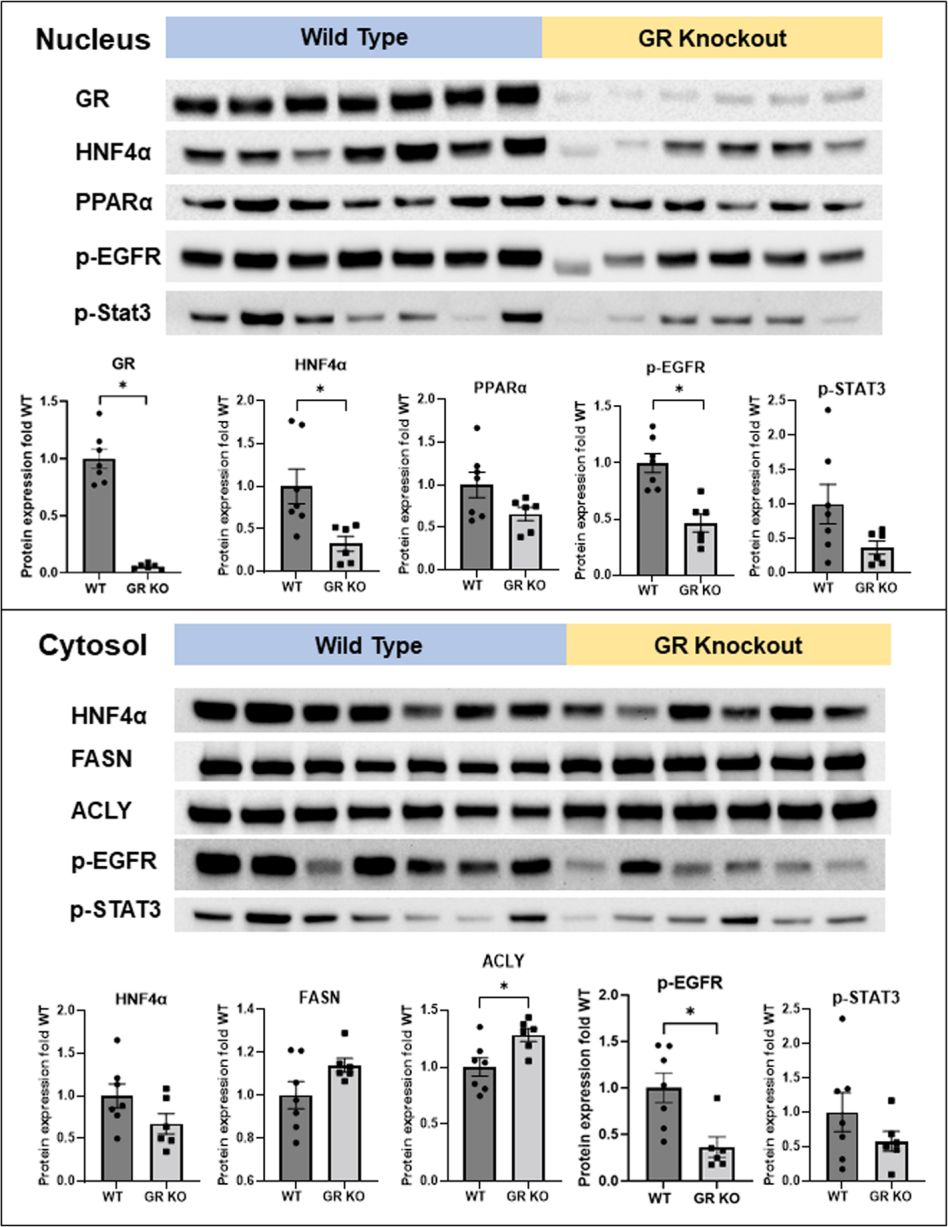
Western blotting quantification of proteins in hepatic nuclear (top) and cytosolic (bottom) extracts in adult male wildtype (WT) and GR knockout (GR KO) mice fed the high-fat-high-sugar diet for 15 d. Band densities were normalized to the loaded total proteins. *N*=6-7 per group, mean ± SE. * *P <* 0.05 versus WT mice

Consistent with the changes in hepatic mRNA expression of GR, Hnf4a, and Acly, GR KO mice had marked decreases in hepatic protein levels of GR (↓95%) and HNF4α (↓67% in the nucleus) and increases of ACLY (↑28%) proteins (Fig. 10). In contrast, hepatic proteins of PPARα tended to be lower (↓34%, *p* = 0.07) in GR KO mice. Currently, it remains unknown the mechanism of the apparent discrepancy between the increased PPARα mRNA and the trend of decreased PPARα protein in the HFHS-fed GR KO mice. Interestingly, hepatic overexpression of Cyp17a1 increases PPARα protein without affecting PPARα mRNA levels [119]. Ligand activation stabilizes PPARα, an unstable protein that is rapidly degraded via the ubiquitin-proteasome pathway [93]. Thus, the trend of decreased PPARα protein in GR KO mice might be due to a decrease of activating ligand or induction of Fat10, an ubiquitin-like protein that promotes proteasomal degradation of proteins. EGFR plays important roles in lipid metabolism, liver injury, and liver regeneration [124]. Activation of signal transducer and activator of transcription 3 (STAT3) by interleukin-6 protects against HFD-fed-induced fatty liver and alcoholic hepatitis [125]. GR KO mice had marked decreases in hepatic phosphorylated EGFR (↓54% in nucleus and ↓64% in cytosol) and a trend of decreases of p-STAT3 (↓63% in nucleus and ↓42% in cytosol) proteins (Fig. 10). Severe steatosis and deficiency of hepatocellular GR impair liver regeneration [126, 127]. Defective activation of EGFR and STAT3 may contribute to impaired liver regeneration due to deficiency of GR in hepatocytes.

## Discussion

In summary, in the HFHS-fed HNF4α HET mice, hepatic down-regulation of HNF4α- and GR-target lipid catabolic genes and induction of lipogenic genes (e.g. Srebp-1c, Fasn, Scd1, and Acly) appear to be responsible for increases of serum and hepatic lipids. HNF4α potently inhibits the transactivation of mouse and human SREBP-1C by LXR. In contrast to the hyperlipidemia in the HNF4α HET mice, induction of the lipogenic genes Acc and Acly as well as down-regulation of genes important for hepatic catabolism and efflux of lipids appear to be responsible for hepatic lipid accumulation and hypolipidemia in the HFHS-fed HNF4α KO mice. Surprisingly, hepatic nuclear GR proteins tended to be decreased in HFHS-fed HNF4α KO mice. In reporter assays, GR acts as a key modulator of HNF4α and PPARα to induce lipid catabolic genes and suppress lipogenic genes. Consistently, HFHS-fed GR KO mice had increased hepatic lipid accumulation, down-regulation of HNF4α and lipid catabolic genes, and induction of lipogenic genes.

The present study suggests a novel key role of hepatic HNF4α in preventing not only the fatty liver but also hyperlipidemia, partly via inhibiting the induction of the key lipogenic enzyme ACC and the master lipogenic factor SREBP-1C. Hepatic induction of ACC in the HNF4α HET mice is likely due to the induction of its transactivator SREBP-1C in these mice [128]. Currently, the mechanism of the strong induction of ACC at both the mRNA and protein levels in the HNF4α KO mice, despite decreased SREBP-1C proteins, remains unclear. Nevertheless, activation of PPARα induces Acc in mouse livers [129]. Thus, loss of HNF4α as a transrepressor and/or the trend of increase of PPARα proteins (Fig. 6) as a transactivator might contribute to the induction of ACC in the HNF4α KO mice. When activated by oxysterols or insulin, LXR bind to LXR response element (LXRE) to transactivate genes. Activation of LXR in the liver causes fatty liver and hyperlipidemia due to increased *de novo* lipogenesis via induction of SREBP-1C [130]. Unsaturated FAs potently down-regulate SREBP-1C to feedback inhibit lipogenesis [131]; how such feedback regulation is compromised in steatosis is poorly understood. Data in this study indicate that HNF4α antagonizes the transactivation of SREBP-1C by LXR to control hepatic lipid metabolism, and thus establishes partial deficiency of HNF4α as a key mechanism of the dysregulation of the two master lipogenic factors LXR and SREBP-1C during hepatosteatosis and hyperlipidemia. In contrast, the marked decrease of hepatic levels of HDCA, a LXR agonist [19], may contribute to the lack of induction of Srebp-1c mRNA in the HNF4α KO mice. Additionally, activation of β-catenin increases insulin sensitivity and strongly inhibits Srebp-1c expression [132, 133]. β-catenin is activated in HNF4α KO mice [134], which might also contribute to the lack in the induction of Srebp-1c mRNA in HNF4α KO mice.

HNF4α deficiency causes dramatic up- and down-regulation of a very large number of genes. Despite extensive studies of how HNF4α transactivates its target genes, little is known about how HNF4α suppresses gene expression. The corepressor SHP can inhibit the transcriptional activities of a large number of transcription factors. Interestingly, HNF4α has been shown to play a critical role in determining the cellular localization of SHP: SHP remains in the cytosol in the absence of HNF4α [135]. The present study found that SHP mediates the inhibition of LXR-activation of SREBP-1C promoter by HNF4α. SHP promotes early fatty liver by inducing PPARγ, whereas loss of SHP aggravates hepatic inflammation and liver cancer [51, 96]. Interestingly, the human SHP promoter is stimulated by HNF4α [136]. The role of SHP in mediating the inhibitory effects of HNF4α on hepatic lipogenesis, inflammation, and liver cancer warrants further investigation.

The present study demonstrates that partial loss of HNF4α results in a moderate elevation of HDL cholesterol and a more marked elevation of LDL/VLDL cholesterol, resulting in an elevated non-HDL/HDL cholesterol ratio (Fig. 1) and CAD risk. Liver-specific overexpression of the Lipase G, Endothelial Type (LIPG) dramatically decreases blood levels of total and HDL-cholesterol by accelerating HDL catabolism and increasing hepatic uptake of HDL-cholesteryl ether [64]. The present study identified a novel gene-dosage-dependent critical role of HNF4α in determining hepatic expression of LIPG in HFHS-fed mice (Fig. 5D). Hepatic expression of LIPC and SR-BI (other genes in HDL-cholesterol uptake) as well as ABCA1, APOA1, and APOA2 (key genes in HDL biogenesis) remain unchanged in the HET mice (Fig. 5D, E), strongly suggesting that hepatic down-regulation of LIPG and the resultant decreased hepatic uptake of HDL-cholesterol may be responsible for the elevated HDL-cholesterol in the HNF4α HET mice. Interestingly, in an animal model of atherosclerosis, LIPG expression is enhanced in the aorta and reduced in the liver of mice developing atherosclerosis [137]. In addition to regulating LIPG-mediated HDL-cholesterol uptake, the present study shows that HNF4α regulates hepatic efflux of lipid and cholesterol by differentially affecting APOC3 and APOA4 expression. In addition to APOB, APOC3 and APOA4 are two of the major apolipoproteins responsible for hepatic secretion of VLDL [138]. In contrast to the association of APOC3 with the small and dense VLDL [139], APOA4 promotes the expansion of larger VLDL proteins that are thought to have less CAD risk [138]. Interestingly, lower serum APOA4 but higher serum APOC3 are associated with an increased risk of CAD [140, 141]. Similar to CAD patients, Apoa4 was lower, whereas Apoc3 tended to be higher, in HFHS-fed HNF4α HET mice than WT mice (Fig. 5D). Taken together, data in this study strongly suggest that partial HNF4α deficiency may be a key driver of not only NAFLD but also hyperlipidemia and CAD risk during HFHS intake.

The present study discovers novel critical roles of HNF4α heteroinsufficiency and KO in regulating hepatic CYP7A1 expression and BA metabolism during HFHS intake. In the KO mice, the dramatic down-regulation of CYP7A1, CYP8B1, and CYP2C70 but a moderate down-regulation of CYP27A1 (59%) will shift the BA biosynthesis from CA to CDCA, resulting in a trend of decreased total CA (↓34%) but a marked 4.9 fold increase of total CDCA. In contrast, the dramatic down-regulation of BAAT is likely responsible for the marked increase of free DCA and decrease of T-DCA in the KO mice. In HNF4α KO mice, the much less decreases in total T-BAs than total sulfated BAs, despite similarly dramatic down-regulation of the conjugating enzymes BAAT and SULT2A8, is likely because most of the sulfated BAs are excreted in the urine without recycling [142], whereas the majority of T-BAs are recycled via enterohepatic circulation. A previous study indicates that the gallbladder bile of HNF4α KO mice has dramatic increases in free BAs (>100 fold) as well as G-DCA and G-CDCA (>20 fold), but only moderate increases of the major taurine-conjugated BAs T-CA, T-MCA, and T-DCA [61]. Hepatic basolateral efflux transporters for BAs are markedly induced in the HNF4α KO mice [12]. Thus, HNF4α deficiency leads to increased basolateral efflux of sulfated BAs for urinary excretion and increased canalicular efflux of free as well as taurine- and glycine-conjugated BAs into the bile to alleviate hepatocyte accumulation of BAs and the resultant liver injury. Currently, the role of CYP7A1 in HFD-induced NAFLD is controversial [143, 144]. Mouse Cyp7a1 promoter has an LXR-binding site and is induced by cholesterol. In contrast, the lack of an LXR site in the human CYP7A1 promoter prevents hepatic induction of human CYP7A1 by high cholesterol, resulting in increased hypercholesterolemia when fed a HFD [143]. Transgenic mice overexpressing CYP7A1 have an expanded BA pool and are resistant to HFD-induced obesity, fatty liver, and insulin resistance [60]. In contrast, Cyp7a1 KO mice are also resistant to HFD-induced obesity, fatty liver, and insulin resistance due to induction of the alternative pathway Cyp27a1 and Cyp7b1 and the resultant increased production of the hydrophilic MCA [144]. However, humans lack the CYP2C70-catalyzed CDCA-MCA pathway and thus have a much more hydrophobic BA profile than rodents [15]. Consequently, CYP7A1 mutation in humans causes hypercholesterolemia, hypertriglyceridemia, and accumulation of cholesterol in the liver, despite induction of the alternative pathway [145]. Thus, the more hydrophilic BAs in livers of HNF4α HET mice (Fig. 4H), despite a marked down-regulation of Cyp7a1 (Fig. 5C), is likely a rodent-specific phenotype. Humans with hepatic HNF4α deficiency may experience a more marked disorder in cholesterol, BA, and lipid metabolism when on HFD than the HFD-fed HNF4α HET mice.

The present study suggests that partial and total deficiency of HNF4α may differentially modulate the BA receptor FXR signaling in the liver. Within the cells, free and taurine-conjugated CA and CDCA have similar potency in activating FXR [146], whereas T-MCAs are FXR antagonists [147]. The dramatic increase of serum and hepatic CDCA, the most potent FXR agonist in HNF4α KO mice (Fig. 4A and Supplemental Figure 2) is consistent with the previous report of activation of FXR in these mice [86]. In contrast, the marked increase of the FXR antagonist T-MCA (Fig. 4E) and the trend of decreases in the FXR agonists DCA and CA (Supplemental Figure 2) in livers of HNF4α HET mice might result in an impaired FXR signaling. It is noteworthy that although the FXR-target gene SHP tended to be induced in both the HNF4α HET and KO mice (Fig. 5B), SHP can also be induced by other nuclear receptor(s). Particularly, LXRα can induce human SHP by binding to the DNA response element in the SHP promoter that overlaps with the previously characterized bile acid response element [148], and the data mining found that LXRα also tends to induce Shp in mouse liver (GSE2644).

Literature suggests that activation of GR in extrahepatic tissues worsens NAFLD, whereas hepatic GR may be protective in lipid metabolism during HFD intake [149–153]. Although many acute effects of GCs mobilize energy and cause weight loss, chronically elevated circulating GCs motivate people to overeat HFHS food and promote obesity and NAFLD via activating GR in extrahepatic tissues [149, 150]. Importantly, circulating GCs markedly increase in mice with liver-specific GR deficiency [153], and literature supports a protective role of hepatic GR against fatty liver during high FA flux [151–153]. In this regard, Stat5/GR double KO mice have more severe steatohepatitis than Stat5 KO mice [153]. Liver-specific overexpression of HSD11B1 in HFD-fed mice down-regulates LXRα and tends to ameliorate steatosis [151]. Importantly, on a HFD, GR +/- mice have higher hepatic TG than WT mice despite similar feed intake [152]. In NAFLD patients, hepatic activities of HSD11B1 decrease, whereas the cortisol-inactivation enzyme increase [22, 154], which may result in local GC deficiency and concomitant activation of the hypothalamic–pituitary–adrenal (HPA) axis to trigger GC release [22, 154]. In contrast, a specific HSD11B1 inhibitor worsens liver fibrosis in mice [155]. The current results from HFHS-fed mice with adult liver-specific KO of GR provide definitive evidence that hepatocellular GR is essential in the protection against HFHS-induced fatty liver. Moreover, hepatic GR may also be important in preventing the development of liver fibrosis, manifested by hepatic induction of PAI-1 and Col1a1 in the GR KO mice. PAI-1 plays a key role in promoting steatosis, inflammation, and fibrosis [121–123]. An increase in circulating PAI-1 mediates GC-induced diabetes and osteopenia in mice [156]. Interestingly, GCs markedly induce PAI-1 in the adipose tissue and muscle, but down-regulates PAI-1 in the liver [156]. Likewise, a strong hepatic induction of PAI-1 in the HFHS-fed GR KO mice was found in this study. In contrast, PPARα induces PAI-1 in mouse liver [157]. Further study on the hepatic GR-PAI-1 pathway in regulating liver fibrosis is warranted.

The present study discovers novel GR-target genes important in the regulation of lipid metabolism. In addition to the known GR-target genes GILZ, MT1, SETDB2, and PAI-1, seven novel GR-target genes were identified, namely SREBP-1C, PPARγ, LCN13, MFSD2A, CYP17A1, G0S2, and FAT10 (Figs. 8A, B and 9). Induction of the two key lipogenic transcription factors SREBP-1C and PPARγ may be responsible for hepatic induction of lipogenic genes and increased lipid accumulation in the GR KO mice. Currently, the mechanism of hepatic suppression of the lipogenic PPARγ by GR is unknown. SREBP-1C is a transactivator [158], whereas GILZ is a transrepressor of PPARγ [159]. Thus, derepression of SREBP-1C or down-regulation of GILZ may lead to the induction of PPARγ in GR KO mice. In hepatocytes, LCN13 directly suppresses hepatic gluconeogenesis and lipogenesis but increases fatty acid β oxidation [160]. Currently, the role of MFSD2A, the key transporter for ω3-fatty acids in fatty liver is still unclear [161]. Given the important role of essential ω3-fatty acids in the protection against fatty liver, the potential role of the GR-MFSD2A pathway in regulating hepatic lipid metabolism warrants further investigation. FAT10 is a 165-amino acid ubiquitin-like protein for ubiquitin-independent proteasomal degradation [162]. GR antagonizes NF-kB [163]. FAT10 is synergistically induced by NF-kB and STAT3, and FAT10 mediates NF-KB activation [164–166]. Interestingly, FAT10-null mice have increased insulin sensitivity and fatty acid oxidation and decreased fat mass [167]. The role of the GR/FAT10 pathway in regulating hepatic protein homeostasis and steatohepatitis warrants further investigation.

The present study demonstrates that hepatic GR has an important role in modulating the transcriptional activity of HNF4α and PPARα during hepatosteatosis. GR and HNF4α cooperatively induce genes that promote lipid catabolism, such as Cyp7a1, Por, SetDb2, and Lcn13, whereas GR antagonizes the activation of the lipogenic Apoc3 by HNF4α. In parallel, treatment of primary human hepatocytes with the GR agonist dexamethasone increases the expression of CYP7A1 and the production of CA [168]. Importantly, this study demonstrates that HNF4α and GR are mutually dependent in the liver: loss of HNF4α tends to decrease nuclear GR proteins, and loss of GR causes marked decreases in mRNA and protein expression of HNF4α.

Activation of the FA receptor PPARα promotes FA catabolism to protect against fatty liver during fasting and HFD intake [27]. However, PPARα can also induce key lipogenic genes [106, 108] and enhance lipogenesis, which may abate its beneficial effects on lipid catabolism. Particularly, PPARα induces the key lipogenic transcription factor Srebp-1c by directly binding to its promoter [106], and induction of lipogenic genes by chronic PPARα activation is abolished in Srebp1 KO mice [169]. Consequently, both protective and aggravating effects of PPARα activation on fatty liver in mice have been reported [27, 108, 129]. Interestingly, GR agonists can antagonize the induction of lipogenic genes by PPARα agonists in mouse hepatocytes [103]. Despite the trend of decreased nuclear PPARα proteins, a group of PPARα-target genes, such as Srebp-1c, Cyp4a14, Cd36, Plin2, Pdk4, Fasn, and G0s2 [170] were induced in GR KO mice (Fig. 9). The present study found that GR and PPARα overlap in their binding to the promoters/introns of these genes (Fig. 8B), and GR antagonizes the induction of SREBP-1C/Srebp-1c, PLIN2, and CD36 by PPARα in reporter assays. The induction of Srebp-1c, Plin2, Cd36, G0s2, and Pdk4 are associated with increased lipid accumulation in HFHS-fed GR KO mice. During fasting, G0s2 is induced by PPARα to promote fat accumulation in the liver [170]. Concurrent activation of LXR and PPARα exacerbates hepatic steatosis in HFD-induced obese mice [171]. Thus, hepatic GR signaling might determine the beneficial versus detrimental effects of PPARα activation on hepatic lipid metabolism, which warrants further investigation. These lipogenic genes Plin2, Pdk4, and Cd36 were also highly elevated in HFHS-fed HNF4α KO mice (Fig. 5). The trend of increased PPARα proteins and decreased GR proteins in HNF4α KO mice (Fig. 6) may contribute to the super-induction of PPARα-target lipogenic genes Plin2, Pdk4, and Cd36. Further in-depth study of liver-specific GR-HNF4α-LXRα and GR-PPARα/PPARγ crosstalk will help understand not only fatty liver but also the progression from simple steatosis to steatohepatitis and cirrhosis.

### Strengths and limitations

The strengths of this study include 1) Use of adult mice with inducible liver-specific heterozygote and knockout of HNF4α to determine the gene-dosage-dependent role of HNF4α in regulating hepatic gene expression and lipid metabolism in HFHS intake; 2) Comprehensive characterization of the phenotype and the underlying mechanism(s) of HFHS-fed HNF4α HET and KO mice by determining hepatic lipids and bile acids, blood chemistry, hepatic mRNA and protein expression, luciferase reporter assays, and data mining of DNA-binding of HNF4α and GR to their target genes; and 3) Use of novel mice with inducible liver-specific knockout of GR to definitively identify the role of hepatic GR in lipid metabolism during HFHS intake.

A major limitation of this study is that only male mice were systematically studied. Compared to males, females are generally protected from fatty liver and hyperlipidemia. It is well known that compared to males, female knockout/heterozygote mice are often protected from genetic deficiency of a given gene. Gender differences in hepatic gene expression due to HNF4α deficiency have been reported: HNF4α deficiency has more profound effects on hepatic gene expression in male mice than female mice [172]. The initial study also included female HNF4α HET and WT mice fed the high-fat-high-sugar diet for 15 days. Surprisingly, HFHS-fed female HNF4α HET mice did not differ from WT mice in hepatic levels of triglycerides, and they had slightly lower hepatic cholesterol than the WT mice (Supplemental Figure 3https://figshare.com/s/910a5b3942600d7a2761). The qPCR data (Supplemental Figure 4https://figshare.com/s/2b1bfb08231c51caab60) showed that these female HET mice had decreased hepatic expression of the bona fide HNF4α-target gene Fitm1 (Fig. 8D), induction of Tweak, and a trend of induction of Srebp1c, similar to the male HET mice. However, these female HET mice did not have induction of key lipogenic genes Scd1, Fasn, Plin2, and Acc1 observed in the male HET mice (Fig. 5). In contrast to the down-regulation of a group of GR-target lipid metabolic genes Cyp7a1, Por, Setdb2, Mt1, Mt2, and Mfsd2a in the male HET mice, these GR-target genes were either unchanged or tended to be induced in female HET mice (Supplemental Figure 4). Moreover, hepatic expression of Fkbp5, a well-established GR-target gene, was significantly induced in these female HET mice. Thus, gender differences in changes in hepatic GR signaling may contribute to the protection of HFHS-fed female HNF4α HET mice from lipid accumulation. Additionally, estrogens play a key role in gender difference in hepatic lipid metabolism [173]. Hepatic mRNA expression of estrogen receptor α (Esr1) remained unchanged in female HET mice (Supplemental Figure 4), although the contribution of estrogen signaling to gender differences in hepatic lipid metabolism during HNF4α partial deficiency can’t be excluded.

## Conclusion

The present study clearly demonstrates that HNF4α and GR play key roles in the protection against HFHS-induced elevation of circulating and hepatic lipids. When the activation of PPARα signaling is impaired by fructose overdose, the cooperative induction of lipid catabolic genes and suppression of lipogenic genes by HNF4α and GR may play a key role in the early resistance to HFHS-induced fatty liver and hyperlipidemia (Graphical abstract). The gene-environment interaction between an obesogenic environment and genetic susceptibility drives the pathogenesis of metabolic disease and NAFLD. Numerous pathological mutations with decreased GC sensitivity have been identified in the human GR gene [174]. Hepatic HNF4α expression is highly variable in humans [175], and HNF4α is markedly decreased in diabetes and NAFLD [4–6]. The missense T130I mutation of HNF4α causes an approximately 50% decrease in the expression of HNF4α protein [176]. Very interestingly, obesity and elevated blood triglycerides are associated with a high frequency of T130I mutation of HNF4α in non-diabetic indigenous Mexicans with high intake of HFHS [177]. Partial deficiency of HNF4α and/or GR due to genetic polymorphism and/or metabolic stresses is thus likely a key mechanism of the loss of resistance to hepatosteatosis and hyperlipidemia during HFHS intake. The identification of partial deficiency of HNF4α and GR as potential key drivers of NAFLD and hyperlipidemia may help develop novel pharmaceutical and dietary interventions for HFHS-induced NAFLD and CAD. Although the activating ligand for HNF4α has not been identified, small activating RNA for HNF4α has been used to successfully induce HNF4α and ameliorate hyperlipidemia and fatty liver in HFD-fed rats [178]. Given GC’s potent anti-inflammatory effects, liver-specific activation of GR may protect against HFHS-induced NAFLD and the progression from NAFLD to NASH and cirrhosis.

## Supplementary Information

**Additional file 1: Supplemental Figure 1.** Pathways of bile acid synthesis and metabolism in mice.

**Additional file 2: Supplemental Table S1.** Primers used for PCR cloning of promoter/intron fragments in the pGL3-basic reporter vectors.

**Additional file 3: Supplemental Table S2.** Primers used for real-time PCR determination of mRNA expression.

**Additional file 4: Supplemental Figure 2.** Serum and hepatic levels of bile acids in adult male wildtype (WT), HNF4α heterozygote (HET), and HNF4α knockout (KO) mice fed 15 d with high-fat-high-sugar diet (HFHS) (*N*=5-6 per group).

**Additional file 5.**

**Additional file 6: Supplemental Figure 3.** Hepatic levels of triglycerides and cholesterol in adult female HNF4α heterozygote (HET) and wildtype (WT) mice fed a high-fat-highsugar diet for 15 d. *N*=6 per group.

**Additional file 7: Supplemental Figure 4.** qPCR determination of hepatic mRNA expression in adult female HNF4α heterozygote (HET) and wildtype (WT) mice fed a high-fat-high-sugar diet for 15 d. *N*=6 per group.

## Data Availability

The data presented in this study are available within the article or in the supplemental files accessible at https://figshare.com or in the public database (GEO DataSets).

## Abbreviations

Abat: 4-aminobutyrate aminotransferase
Abca1: ATP binding cassette subfamily A member 1
Abcg8: ATP binding cassette subfamily G member 8
Acc1: Acetyl-CoA carboxylase 1
Acc2: Acetyl-CoA carboxylase 2
Acly: ATP citrate lyase
Acot1: Acyl-CoA thioesterase 1
Acox1: Acyl-coenzyme A oxidase 1
Alas1: Aminolevulinic acid synthase 1
Apoa2: Apolipoprotein A2
Apoa4: Apolipoprotein A4
Apob: Apolipoprotein B
Apoc1: Apolipoprotein C1
Apoc3: Apolipoprotein C3
Avpr1a: Arginine VP receptor 1A
BA: Bile acid
Baat: Bile acid-CoA:amino acid N-acyltransferase
Bsep: Bile salt export pump
C8orf4: Transcriptional and immune response regulator
CA: Cholic acid
CAD: Cardiovascular disease
Ccl2: C-C motif chemokine ligand 2
Ccnd1: Cyclin d1
Cd36: Fatty acid translocase
CDCA: Chenodeoxycholic acid
ChIP: Chromatin immunoprecipitation
Chrebpb: Carbohydrate-responsive element-binding protein beta
Cited2: Cbp/p300-interacting transactivator 2
Col1a1: Collagen 1a1
Cxcl1: C-X-C motif ligand 1
Cyp27a1: Cytochrome P450 27a1
Cyp2c70: Cytochrome P450 2c70
Cyp4a14: Cytochrome P450 4a14
Cyp7a1: Cytochrome P450 7a1
Cyp7b1: Cytochrome P450 7b1
Cyp8b1: Cytochrome P450 8b1
DCA: Deoxycholic acid
EGFR: Epidermal growth factor receptor
Fasn: Fatty acid synthase
Fat10 (Ubd): HLA-F-adjacent transcript 10
Fgf21: Fibroblast growth factor 21
Fitm1: Fat storage-inducing transmembrane protein 1
Fitm2: Fat storage-inducing transmembrane protein 2
Fn14: Fibroblast growth factor-inducible 14
Fxr: Farnesoid X receptor
G0s2: G0/G1 switch gene 2
G6pc: Glucose-6-phosphatase catalytic subunit
GABA: γ-Aminobutyric Acid
Gale: UDP-galactose-4-epimerase
Gcgr: Glucagon receptor
GCs: Glucocorticoids
Gilz: Glucocorticoid-induced leucine zipper
GR: Glucocorticoid receptor
HCA: Hyocholic acid
HDCA: Hyodeoxycholic acid
HET: Heterozygote
HFD: High-fat diet
HFHS: High-fat-high-sugar diet
HI: Hydrophobicity index
Hmgcr: HMG-CoA reductase
Hmgcs2: Hydroxymethylglutaryl-CoA synthase
Hnf1b: Hepatocyte nuclear factor 1B
Hnf4a: Hepatocyte nuclear factor 4A
Hnf4g: Hepatocyte nuclear factor 4G
HSD11B1: 11β-hydroxysteroid dehydrogenase type 1
Il1r1: Interleukin-1 receptor 1
KO: Knockout
LCA: Lithocholic acid
Lcn13: Lipocalin 13
Ldlr: Low-density lipoprotein receptor
Lipc: Lipase C, Hepatic Type
Lipe: Lipe lipase E, hormone sensitive type
Lipg: Lipase G, Endothelial Type
Lpl: Lipoprotein lipase
Lxra: Liver X receptor alpha
MCA: Muricholic acid
Mcad: Medium-chain acyl-CoA dehydrogenase
Mfsd2a: Major facilitator super family domain containing 2a
Mt1: Metallothionein-1
Mt2: Metallothionein-2
Mttp: Microsomal triglyceride transfer protein
Myc: MYC Proto-Oncogene
NAFLD: Non-alcoholic fatty liver disease
NASH: Non-alcoholic steatohepatitis
Nrf2: NF-E2-related factor-2
Ntcp: Na+-taurocholate cotransporting polypeptide
Oatp1b2: Organic anion transporting polypeptide 1b2
p21: Cyclin Dependent Kinase Inhibitor 1A
PAI-1: Plasminogen activator inhibitor-1
Pck1: Phosphoenolpyruvate carboxykinase 1
Pcsk9: Proprotein convertase subtilisin/kexin type 9
Pde9a: Phosphodiesterase 9A
Pdk4: Pyruvate dehydrogenase kinase 4
Pgc1a: PPARγ coactivator 1-alpha
Pgc1b: PPARγ coactivator 1-beta
Pgk1: Phosphoglycerate Kinase 1
Pklr: Liver-type pyruvate kinase
Plin2: Perilipin 2
Por: Cytochrome P450 reductase
Ppara: Peroxisome proliferator activated receptor A
Ppard: Peroxisome proliferator activated receptor D
Pparg: Peroxisome proliferator activated receptor G
Pptc7: Protein Phosphatase Targeting COQ7
Prdx1: Peroxiredoxin 1
Sc5d: Lathosterol 5-desaturase
Scd1: Stearoyl-CoA desaturase-1
Setdb2: SET Domain Bifurcated Histone Lysine Methyltransferase 2
Shp: Small heterodimer partner
Sox9: SRY-Box Transcription Factor 9
SR-BI: Scavenger receptor class B, type I
Srebp1c: Sterol regulatory element binding transcription factor 1C
Srebp2: Sterol regulatory element binding transcription factor 2
STAT3: Signal transducer and activator of transcription 3
Sult2a8: Sulfotransferase 2a8
T-: Taurine-
TG: Triglycerides
Tgfb1: Transforming growth factor beta1
Tweak: TNF-like weak inducer of apoptosis
UDCA: Ursodeoxycholic acid
Vldlr: VLDL receptor
WT: Wildtype
