# Supplementary figures and images for: Crosstalk of hepatocyte nuclear factor 4a and glucocorticoid receptor in the regulation of lipid metabolism in mice fed a high-fat-high-sugar diet
Source: Lipids Health Dis. 2022 May 25;21:46. doi: 10.1186/s12944-022-01654-6 (PMC9134643; doi:10.1186/s12944-022-01654-6)

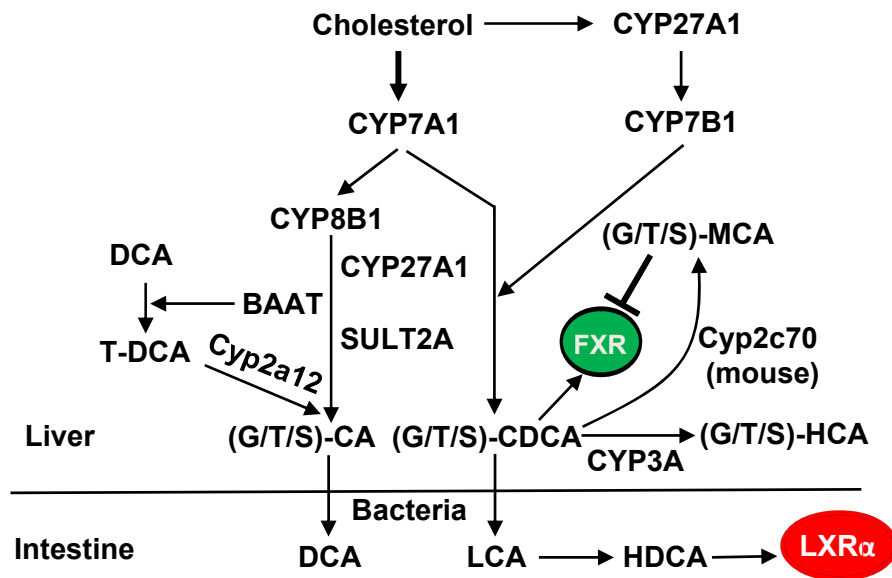

Pathways of bile acid synthesis and metabolism in mice.

Supplement: Supplementary file 1 — Additional file 1: Supplemental Figure 1. Pathways of bile acid synthesis and metabolism in mice. [file 12944_2022_1654_MOESM1_ESM.pdf]
