# Supplementary material for: Crosstalk of hepatocyte nuclear factor 4a and glucocorticoid receptor in the regulation of lipid metabolism in mice fed a high-fat-high-sugar diet
Source: Lipids Health Dis. 2022 May 25;21:46. doi: 10.1186/s12944-022-01654-6 (PMC9134643; doi:10.1186/s12944-022-01654-6)
Supplement: Supplementary file 2 — Additional file 2: Supplemental Table S1. Primers used for PCR cloning of promoter/intron fragments in the pGL3-basic reporter vectors. [file 12944_2022_1654_MOESM2_ESM.docx]

**Supplemental table S1. Primers used for PCR cloning of promoter/intron fragments in the pGL3-basic reporter vectors.**

| **Gene name** | **Forward primer** | **Reverse primer** | **Product (bp)** |
| --- | --- | --- | --- |
| **Mouse** |  |  |  |
| Lcn13 | AAAAAGGTACCAGGTGAGCTGAGGCCAGAAG | AAAAATACGCGTGGTCATGAGTATGTGTCCTCT | 445 |
| Setdb2 | TATAAGGTACCCCTGTTTCATAACTCCCCAAAG | TTTGTACGCGTCGTTTCTACTACCTTCCTCAG | 357 |
| Mfsd2a | AAAACGGTACCTACCTGCCCCAGGAACCACA | AATCAACGCGTCTTCTCAAAGTACACACAGTAG | 369 |
| Por | ATTCTGGTACCCTTTGGAGGTTGAAGCAGGAG | AAAAAAACGCGTGTACCAAAGTCCATCTTGGC | 813 |
| Alas1 | AAAAGGTACCGTGTTCAGACTGTAGCCAGTG | AAATACGCGTTGGAAAGTCACACTTCAAAGCT | 230 |
| Cyp7a1 | CTCAGGTACCCCTTTAGGATCGGTTGCTGTA | AAAAACGCGTCAAAAGCAGGAAAACGTCCCAA | 520 |
| Apoc3 | TTTTGGTACCCCTTCATCCTCACCTTCTTTG | TTTAACGCGTAGGGGCATTACCTGGAGTAG | 755 |
| Fitm1 | AAAAAGGTACCAGTCCGAGCACTGCTGGGAT | AATTAACGCGTGTTGAAGAAGTTGCCGTGGCT | 217 |
| **Human** |  |  |  |
| POR | AAAAAGGTACCAGGTGAAAGTTAAGAACAGTGC | TTCAAACGCGTAACCAACAGTGGCTTTTCTCC | 450 |
| SETDB2 | AAAAGGTACCCACGATCTTTGCTCACTACAAC | AAAAACGCGTCATAAGCTCACTGTGACCTTG | 791 |
| CD36 | TAAAGGTACCCAGCACATGGATTGAAGTTTTG | AAAAACGCGTAGCCCTTCTCCAGATTCTATG | 477 |
| PLIN2 | AATTGGTACCTTGGGACCGCTGCTCTGCTC | AATTACGCGTTTCCCTTTCGATAATGTCCCT | 272 |
