# Supplementary material for: Crosstalk of hepatocyte nuclear factor 4a and glucocorticoid receptor in the regulation of lipid metabolism in mice fed a high-fat-high-sugar diet
Source: Lipids Health Dis. 2022 May 25;21:46. doi: 10.1186/s12944-022-01654-6 (PMC9134643; doi:10.1186/s12944-022-01654-6)
Supplement: Supplementary file 3 — Additional file 3: Supplemental Table S2. Primers used for real-time PCR determination of mRNA expression. [file 12944_2022_1654_MOESM3_ESM.docx]

**Supplemental table S2. Primers used for real-time PCR determination of mRNA expression.**

| **Gene** | **Forward** | **Reverse** |
| --- | --- | --- |
| Abat | AGCTGGAGACTTGCATGGTTA | ATTGCTTTGGAGTGTGTGGTC |
| Abca1 | CTCTCAGACAACACTTGACCA | GACCTCGCTCTTCCTTCCTT |
| Abcg8 | CAACAGTCTGTACTTCACCTAC | CTGATTTCATCTTGCCACCGT |
| Acc1 | AGAAACCCGAACAGTGGAACT | AGGTAGCCCTTCACGGTTAAA |
| Acc2 | CAAACAGATATTCCAAGTGGCT | CTGGGAACTGATCTGGGTGTA |
| Acly | CCCAGTGAACAACAGACCTAT | ACAAAGATGGTGACCTCATGC |
| Acot1 | GAAGAAGCCGTGAACTACCT | GATTTCTCAGGATAGTCACAGG |
| Acox1 | CACAGGAAGAGCAAGGAAGTG | CCATAGAGAGAATATAAGAGACAC |
| Alas1 | CAGCACCAGCGAAATGTCAAG | ATGCCTGGTCATCAACTCATC |
| Apoa2 | CCAAGACCTCAGAGATTCAGA | CGGTTTCTCCTCAAGGTTCAT |
| Apoa4 | TGCCAACAAAGTAACCCAGAC | CACATTCTCCTTGATCGTGGT |
| Apob | CTGCTGCTGTTCCTGCTACT | CCACAGATTTGGGGGACCTC |
| Apoc1 | CGCTCTTCCTGTCCTGATTGT | CGGGTCTTGGTCAAAATTTCC |
| Apoc3 | ACAAGCCTCCAAGACGGTCCA | AGGGTTAGAATCCCAGAAGCC |
| Avpr1a | GTCAGTCTGGGATACCAATTTC | GCTCATGCTATCCGAGTCATC |
| Baat | TGAGGGTTGCTGTAAAACTACT | CAGGTTTCAAGGACCAGAAAAG |
| Bsep | GGTAAAAGCACCAGCATCCAG | AGTCAAATAACACGGGCTCCT |
| C8orf4 | CAGCATGTCCTCGTCTCTTC | AGGGACTCCTGGTCTATGTTC |
| Ccl2 | GTGTCCCAAAGAAGCTGTAGTT | GCTGAAGACCTTAGGGCAGAT |
| Ccnd1 | CGGATGAGAACAAGCAGACC | ACTCTGGAAAGAAAGTGCGTTG |
| Cd36 | CATATTGGTCAAGCCAGCTAG | AGCAACAAACATCACCACTCC |
| Chrebpb | GTGGAGCTCAGTGGCAAGC | CTTGTCCCGGCATAGCAAC |
| Cited2 | ATGGGCGAGCACATACACTAC | GAACTGGGAGTTGTTAAACCTG |
| Col1a1 | TGATGCTAACGTGGTTCGTG | CGATCCAGTACTCTCCGCTCT |
| Cxcl1 | CTGGGATTCACCTCAAGAACA | CGTTACTTGGGGACACCTTTT |
| Cyp17a1 | ATCATCAATCTCTGGGCACTG | AACTGGGTGTGGGTGTAATGA |
| Cyp27a1 | CTATGGGATCTTCATCGCACA | CTCAGGAATGGAGGGTTTCAG |
| Cyp2c70 | ACTGTATACCTGGGCATGAAG | GCCGGGTTTGTTTCCATGTTT |
| Cyp4a14 | CAACATCATCTACAATATGTCCTC | AGCTGTTCCTATCCTCCATTCT |
| Cyp7a1 | TGTGGTAGTGAGCTGTTGCAT | CCCATCAGTTTGCAGGTAAAA |
| Cyp7b1 | CGGAAATCTTCGATGCTCCAA | AATCGGGGTGCTGAATACCTAA |
| Cyp8b1 | AGTTGCAGCGTCTCTTCCAT | CCTTGCTCCCTCAGAAACTG |
| Elovl6 (FAE) | CTCAGCAAAGCACCCGAACTA | ATCTGGGACAAGGTGATGAAC |
| Fasn | GACCTTCATGGACACAATGCT | CGTTGTCACATCAGCCACTTG |
| Fat10 (Ubd) | ATGGCTTCTGTCCGCACCTG | TTTTGGAGTCTAGCAGAAGGAT |
| Fgf21 | TGAAGCCCACCTGGAGATCA | TCAAAGTGAGGCGATCCATAG |
| Fitm1 | TACACTGTCTCCTCCCACAC | GAGTAGGAAAACAAGACGCAGT |
| Fitm2 | CCTTACCAACTACCACCTGAC | AATGTTGGAGAAGAGGGCTGT |
| Fn14 | AGCAAGCACCAGGCACCTC | TCCCAGGCAGAAGTCGCTGT |
| Fxr | GCAGACCTGTTGGAAGAAAGA | TGTCTGGAGAGAGGATGACGA |
| G0s2 | AAGAACGCCAAAGCCAGTCT | TCTGCGCCATCATCTCCTTG |
| G6pc | AGTCTTGTCAGGCATTGCTGT | AAGAGGGTTCCCAGGTTTTTG |
| Gale | GTATTCCCAACAACCTCATGC | TGTAATCCCTCACACCTGTCC |
| Gcgr | CCACCTACTGAGCTGGTCTG | GCAGGAAATGTTGGCAGTGG |
| Gilz | CACCCTTGGAGTCACTTCTCT | CCGCTATAGGATAGGCTTTGG |
| GR_exon3 | GCCGCTCAGTGTTTTCTAATG | TGCTGTCCTTCCACTGCTCT |
| Hmgcr | TGGAGAATGCAGAGAAAGGTG | GATGGGCATATATCCGATCAC |
| Hmgcs2 | ACCTGCTCACCTGCTCTCA | GTTCCATCAGCCTCTGTACCA |
| Hnf1b | AGAGGGAGGTGGTCGATGTCA | AGCTCTGGACTGTCTGGTTGA |
| Hnf4a (exon4) | AGAAGGAAGCTGTCCAAAATGA | TGCCCGAATGTCGCCATTGAT |
| Hnf4g | GGAGAACATCTGCTGCTTGGA | GCTCATCGAGAACTCTGTTGG |
| Igfbp1 | CCTGCCAACGAGAACTCTATAA | CCAGGGATTTTCTTTCCACTC |
| Il1b | CCAAAAGATGAAGGGCTGCT | AAGAAGGTGCTCATGTCCTCA |
| Il1r1 | ATGAGTTACCCGAGGTCCAGT | TAGTCCCCTCTGTGCTCTTCA |
| Il6ra | GTATCAGCCATACCTGGAAGG | TGGTCCTGGGCTCTGCTATC |
| Lcn13 | CGGGATGGGAAAGCTCATGG | TTATCTCCGATCTCTGGCACA |
| Ldlr | AAGGCTGTGGGCTCCATAGG | GGTCCTCACTGATGATGGTGT |
| Lipc | GGCATAGAACCTCACCACTC | TTTGCATGGGTCTCTTGACTC |
| Lipe | CTGGAACTAAGTGGACGCAAG | TCTCCATTGACTGTGACATCTC |
| Lipg | TCGGCTTTTGGAGCGTCTAT | TGGTACTCCAGTGGGTTTATG |
| Lpin1 | TGTTCAAGAGACTGACAACGAT | AGGCACCTGATTCTGTCTACA |
| Lpl | GAAACCAGTAGGGCATGTTGA | TCAGCAGGGAGTCAATGAAGA |
| Lxra | TCCGTGCAGGAGATTGTTGAC | TCAAAGATGGGGTTGATGAACT |
| Mcad | GTTGACGGAACAGCAGAAAGA | ACCCATACGCCAACTCTTCG |
| Mfsd2a | TCGATGACTTCCACCTGAAAC | CTCTGGTAGTTGGCAAAGTCG |
| Mt1 | CCCAACTGCTCCTGCTCCA | GGTAGAAAACGGGGGTTTAGT |
| Mt2 | ATGGACCCCAACTGCTCCTG | CACTTGTCGGAAGCCTCTTTG |
| Mttp | GGTCTGGATTTACAACGGCAA | CCTGCTATGGTTTGTTGGAAG |
| Myc | AGCTCGCCCAAATCCTGTAC | TCTCCACAGACACCACATCAA |
| Nrf2 | GATAGTGCCCCTGGAAGTGT | TCGTGTGAGATGAGCCTCTAA |
| Ntcp | GCCATGAAGGGGGACATGAA | TGCCTTGAGGACGTAGGGTA |
| Oatp1b2 | TATTCTGACTGCGTTGCCACA | CCCTTTTCACAACCTTTCTCC |
| p21 | TGTCTTGCACTCTGGTGTCTG | GGCACTTCAGGGTTTTCTCTT |
| Pai1 | TCACTTTACCCCTCCGAGAAT | GAGCTGTGCCCTTCTCATTG |
| Pck1 | CATCACCTCCTGGAAGAACAA | CTACGGCCACCAAAGATGATA |
| Pcsk9 | GGAACCTACATTGTGGTGCTG | ACTCCACATGGGGCAACTTCA |
| Pde9a | CAAAGTGACCAAAGCAACAGC | TCTTCTGCAACTCCTTCATGG |
| Pdk4 | CAGAAGACCAGAAAGCCCTGT | TTTCCCGTCTTTGAGTCACTG |
| Pgc1a | ATCACGTTCAAGGTCACCCTA | CAAAGCGGTCTCTCAATTCTG |
| Pgc1b | ATGTGCCTGGATACAGAGACC | CCAAGAGAGTCGCTTTGTGAC |
| Pgk1 | CCCAGAAGTCGAGAATGCCT | CTCGGTGTGCAGTCCCAAAA |
| Pklr | AGCAAAATCGAGAACCATGAAG | CCAAGAAAACCTTCTCTGCTG |
| Plin2 | CAACTATGAACGGCTGGAGTC | ATTCAATCAGGTGGACAGTGG |
| Por | CACATCCTAGCCATTCTCCAA | ACTTCGCTTCATACTCCACAG |
| Ppara | CTGCCTTCCCTGTGAACTGA | TGGGGAGAGAGGACAGATGG |
| Ppard | CCACAACGCACCCTTTGTCA | GAGGAAGAGGCTGCTGAAGTT |
| Pparg | CCCAATGGTTGCTGATTACAAA | GAGGGAGTTAGAAGGTTCTTC |
| Pptc7 | TCAACACTCCATTCCAGCTCT | CATCTGTTGCTGTCAGGATGA |
| Prdx1 | ATGCCAGATGGACAATTCAAAG | CGGCTCTATCACTGAAAGCAA |
| Sc5d | GCCGCCGATTACTACTTCTTC | AATAGCTGAGGGTTGCACAGA |
| Scd1 | TAGCTTTGGGTGCCTTATCTC | ACGAGGACGACAATACAATCG |
| Setdb2 | CATTACAGTGAGAAAAACATGGG | CTGGGTTTGAACTTAAGTCCTC |
| Shp | ACGATCCTCTTCAACCCAGAT | GATGTCAACGTCTCCCATGAT |
| Sox9 | TGAAGAAGGAGAGCGAGGAAG | GCTCTCGTTCAGCAGCCT |
| SR-BI | ACCTTCAATGACAACGACACC | CCACAGGATCTCACCAACTG |
| Srebp1c | ACGGAGCCATGGATTGCACA | CTGTCTCACCCCCAGCATAG |
| Srebp2 | GCTGGTTTGACTGGATGGTTC | AAGCAGGTTTGTAGGTTGGCA |
| Sult2a8 | CCTGGGAGAGAATTTGGAATC | TTCAGCCTGGGCTACTGTAAA |
| Tgfb1 | CGCCATCTATGAGAAAACCAAA | TCAAAAGACAGCCACTCAGG |
| Tweak | GAATCCCCAGACAGAGGAAAG | CATAATGGGCTGCAATAGCTC |
| Vldlr | CGACCTGACCAGTTTGAATGT | GGCACTGATTGACGTTTTTGC |
