# Supplementary material for: Crosstalk of hepatocyte nuclear factor 4a and glucocorticoid receptor in the regulation of lipid metabolism in mice fed a high-fat-high-sugar diet
Source: Lipids Health Dis. 2022 May 25;21:46. doi: 10.1186/s12944-022-01654-6 (PMC9134643; doi:10.1186/s12944-022-01654-6)
Supplement: Supplementary file 4 — Additional file 4: Supplemental Figure 2. Serum and hepatic levels of bile acids in adult male wildtype (WT), HNF4α heterozygote (HET), and HNF4α knockout (KO) mice fed 15 d with high-fat-high-sugar diet (HFHS) (N=5-6 per group). [file 12944_2022_1654_MOESM4_ESM.pdf]

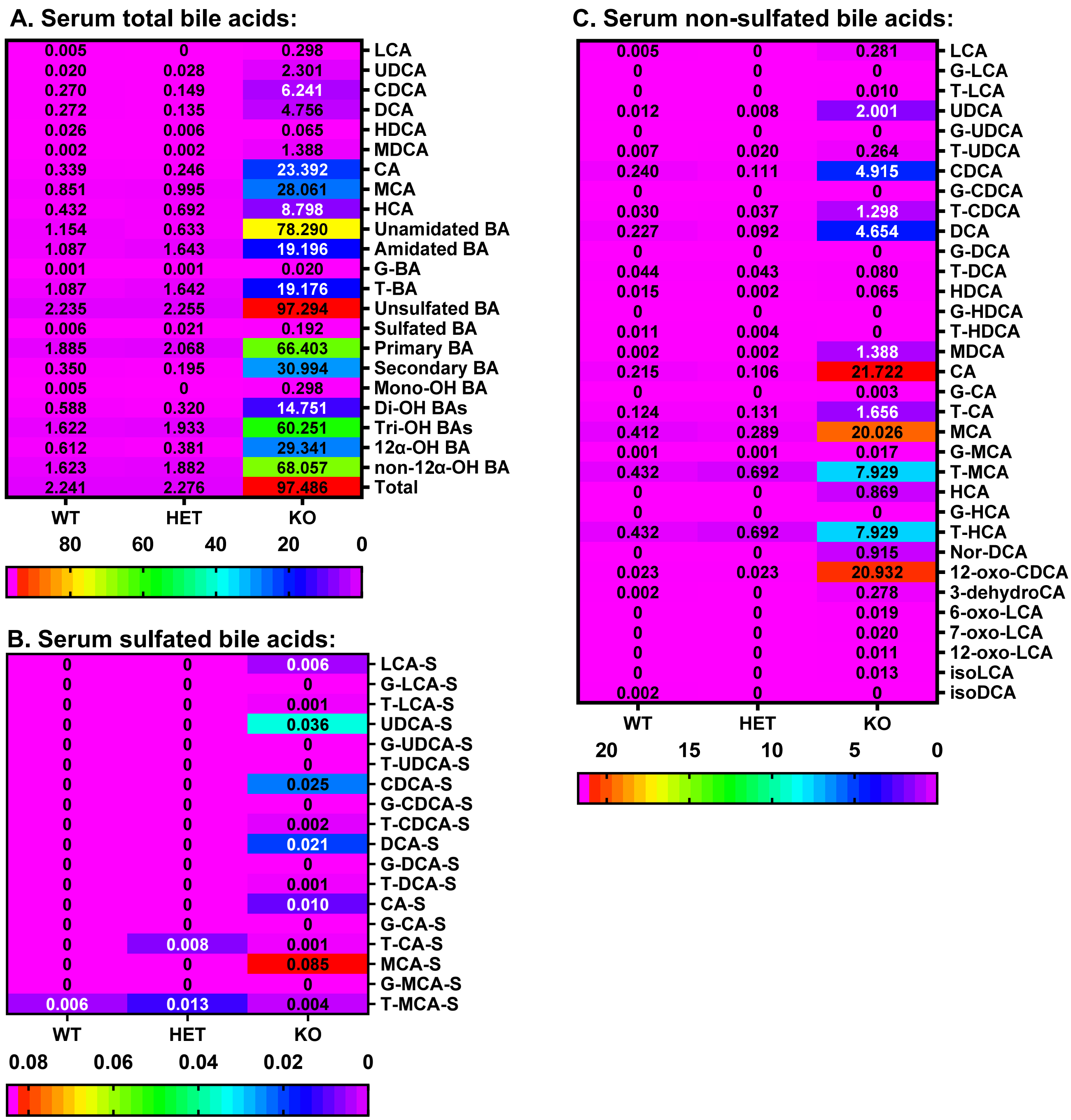

Supplemental Figure 2. Serum and hepatic levels of bile acids in adult male wildtype (WT), HNF4α heterozygote (HET), and HNF4α knockout (KO) mice fed 15 d with high-fat-high-sugar diet (HFHS) (N=5-6 per group). Bile acids were quantified by LC-MS/MS. (**A**) serum levels of total bile acids; (**B**) serum levels of sulfated bile acids; (**C**) serum levels of non-sulfated bile acids. The numbers in the heat maps represent mean bile acid concentrations (μM).

D. Hepatic total bile acids:

	WT	HET	KO
LCA	0.15	0.07	0.70
UDCA	2.68	3.44	3.05
CDCA	3.98	4.50	23.54
DCA	12.28	7.50	5.33
HDCA	1.77	1.48	0.25
MDCA	2.80	2.69	3.35
CA	69.12	61.72	46.00
MCA	79.56	85.87	187.70
HCA	0.08	0.05	1.36
Unamidated BA	119.91	130.65	318.03
Amidated BA	78.14	79.12	37.85
G-BA	0.20	0.22	0.06
T-BA	77.94	78.90	37.80
Unsulfated BA	177.70	191.49	355.19
Sulfated BA	20.35	18.28	0.70
Primary BA	144.14	144.45	258.37
Secondary BA	45.30	57.63	97.29
Mono-OH BA	0.15	0.07	0.70
Di-OH BAs	23.51	19.62	35.53
Tri-OH BAs	148.77	147.64	235.06
12α-OH BA	81.49	69.26	51.46
non-12α-OH BA	107.96	132.81	304.20
Total	198.05	209.77	355.89

Color scale: 300 200 100

E. Hepatic sulfated bile acids:

	WT	HET	KO
LCA-S	0	0	0.02
G-LCA-S	0	0	0
T-LCA-S	0.01	0	0.01
UDCA-S	0.05	0.05	0.05
G-UDCA-S	0.01	0.01	0.01
T-UDCA-S	0	0	0
CDCA-S	0.01	0.01	0.05
G-CDCA-S	0	0	0
T-CDCA-S	0.09	0.15	0
DCA-S	0.02	0.01	0.04
G-DCA-S	0	0	0
T-DCA-S	0.39	0.33	0.01
CA-S	0.71	0.25	0.13
G-CA-S	0.01	0.01	0
T-CA-S	10.44	9.77	0.15
MCA-S	0.18	0.08	0.07
G-MCA-S	0	0.01	0
T-MCA-S	8.43	7.60	0.16

Color scale: 10 5 0

F. Hepatic non-sulfated bile acids:

	WT	HET	KO
LCA	0.04	0.02	0.55
G-LCA	0	0	0
T-LCA	0.11	0.05	0.12
UDCA	2.05	2.48	2.57
G-UDCA	0.01	0.01	0
T-UDCA	0.56	0.89	0.41
CDCA	2.55	2.71	14.16
G-CDCA	0.01	0.01	0
T-CDCA	1.32	1.63	9.33
DCA	1.08	0.50	4.27
G-DCA	0.01	0.01	0
T-DCA	10.77	6.65	1.00
HDCA	1.75	1.28	0.22
G-HDCA	0	0	0
T-HDCA	0.02	0.20	0.04
MDCA	2.80	2.69	3.35
CA	14.53	9.16	37.66
G-CA	0.10	0.09	0.01
T-CA	43.34	42.44	8.06
MCA	68.46	68.93	168.97
G-MCA	0.05	0.07	0.03
T-MCA	2.44	9.17	18.47
HCA	0.06	0.04	1.31
G-HCA	0	0	0
T-HCA	0.02	0.01	0.04
Nor-DCA	0.01	0.01	0.01
12-oxo-CDCA	23.74	40.84	83.94
3-dehydroCA	0.08	0.04	0.11
6-oxo-LCA	0.04	0.06	0.04
7-oxo-LCA	0.02	0	0.02
12-oxo-LCA	0.01	0.03	0.01
isoLCA	1.72	1.47	0.46
isoDCA	0	0	0

Color scale: 150 100 50 0

Supplemental Figure 2. Serum and hepatic levels of bile acids in adult male wildtype (WT), HNF4α heterozygote (HET), and HNF4α knockout (KO) mice fed 15 d with high-fat-high-sugar diet (HFHS) (N=5-6 per group). Bile acids were quantified by LC-MS/MS. (**D**) hepatic levels of total bile acids; (**E**) hepatic levels of sulfated bile acids; (**F**) serum levels of non-sulfated bile acids. The numbers in the heat maps represent mean bile acid concentrations (μM).
