# Supplementary material for: Crosstalk of hepatocyte nuclear factor 4a and glucocorticoid receptor in the regulation of lipid metabolism in mice fed a high-fat-high-sugar diet
Source: Lipids Health Dis. 2022 May 25;21:46. doi: 10.1186/s12944-022-01654-6 (PMC9134643; doi:10.1186/s12944-022-01654-6)
Supplement: Supplementary file 6 — Additional file 6: Supplemental Figure 3. Hepatic levels of triglycerides and cholesterol in adult female HNF4α heterozygote (HET) and wildtype (WT) mice fed a high-fat-highsugar diet for 15 d. N=6 per group. [file 12944_2022_1654_MOESM6_ESM.pdf]

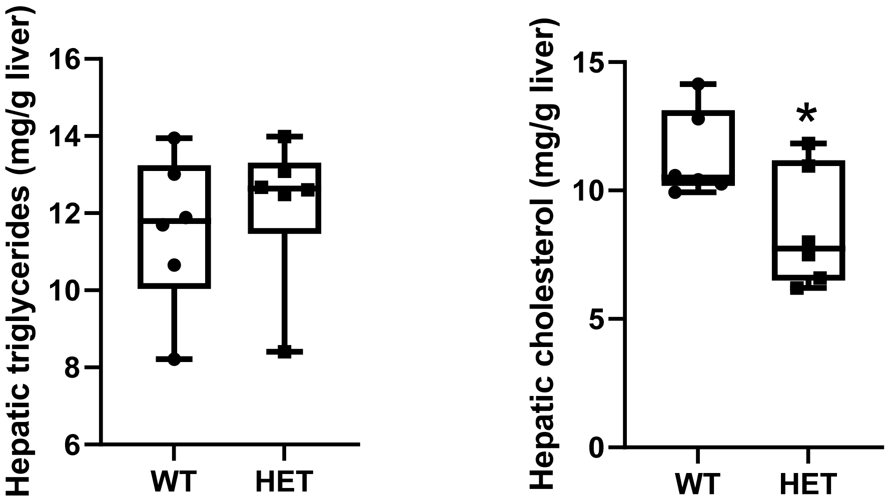

Supplemental Figure 3. Hepatic levels of triglycerides and cholesterol in adult female HNF4α heterozygote (HET) and wildtype (WT) mice fed a high-fat-high-sugar diet for 15 d. N=6 per group. In the box plots, the end of the lower whisker is the minimum value, the boundary of the box closest to zero indicates the 25 th percentile, a black line within the box marks the median, the boundary of the box farthest from zero indicates the 75 th percentile, and the end of the upper whisker is the maximum value. * $p < 0.05$ versus WT group.
